# An overview of natural products that modulate the expression of non-coding RNAs involved in oxidative stress and inflammation-associated disorders

**DOI:** 10.3389/fphar.2023.1144836

**Published:** 2023-04-24

**Authors:** Jubilate Afuoti Ngum, Fabrice Junior Tatang, Michelle Hako Toumeni, Sarah Ngate Nguengo, Ulrich Stephane Fotso Simo, Cybelle Fodieu Mezajou, Charleine Kameni, Natacha Njike Ngongang, Maxwell Fofou Tchinda, Fabrice Fabien Dongho Dongmo, Mazarin Akami, Annie Rosalie Ngane Ngono, Ousman Tamgue

**Affiliations:** Department of Biochemistry, Faculty of Sciences, University of Douala, Douala, Cameroon

**Keywords:** oxidative stress, inflammation, disorders, non-coding RNAs, natural products

## Abstract

Oxidative stress is a state in which oxidants are produced in excess in the body’s tissues and cells, resulting in a biological imbalance amid the generation of reactive oxygen and nitrogen species (RONS) from redox reactions. In case of insufficient antioxidants to balance, the immune system triggers signaling cascades to mount inflammatory responses. Oxidative stress can have deleterious effects on major macromolecules such as lipids, proteins, and nucleic acids, hence, Oxidative stress and inflammation are among the multiple factors contributing to the etiology of several disorders such as diabetes, cancers, and cardiovascular diseases. Non-coding RNAs (ncRNAs) which were once referred to as dark matter have been found to function as key regulators of gene expression through different mechanisms. They have dynamic roles in the onset and development of inflammatory and oxidative stress-related diseases, therefore, are potential targets for the control of those diseases. One way of controlling those diseases is through the use of natural products, a rich source of antioxidants that have drawn attention with several studies showing their involvement in combating chronic diseases given their enormous gains, low side effects, and toxicity. In this review, we highlighted the natural products that have been reported to target ncRNAs as mediators of their biological effects on oxidative stress and several inflammation-associated disorders. Those natural products include Baicalein, Tanshinone IIA, Geniposide, Carvacrol/Thymol, Triptolide, Oleacein, Curcumin, Resveratrol, Solarmargine, Allicin, aqueous extract or pulp of Açai, Quercetin, and Genistein. We also draw attention to some other compounds including *Zanthoxylum bungeanum*, Canna genus rhizome, Fuzi-ganjiang herb pair, *Aronia melanocarpa*, Peppermint, and Gingerol that are effective against oxidative stress and inflammation-related disorders, however, have no known effect on ncRNAs. Lastly, we touched on the many ncRNAs that were found to play a role in oxidative stress and inflammation-related disorders but have not yet been investigated as targets of a natural product. Shedding more light into these two last points of shadow will be of great interest in the valorization of natural compounds in the control and therapy of oxidative stress- and inflammation-associated disorders.

## 1 Introduction

Oxidative stress (OS) is a state in which excess oxidants are produced in the body’s tissues and cells, resulting in a biological inequality between the generation of (RONS) from redox reactions and the antioxidant defense system. OS comes into effect in the body when there is an imbalance between free radicals and antioxidants (Michael, 2020). Reactive oxygen species (ROS) can be generated from oxygen radicals like Superoxide radicals (O_2_^•−^), hydroxyl radicals (•OH), peroxyl (RO_2_^.^), and alkoxyl (RO-) as well as non-radical molecules which are easily oxidized like ozone (O_3_), hydrogen peroxide (H_2_O_2_, singlet oxygen (^1^O_2_), hypochlorous acid (HOCL), (Gabriele et al., 2017) formed during normal aerobic metabolism in the body. They are generated from sources like the mitochondria, cytosol, plasma membranes, and peroxisomes and are important for tissue and cell physiological balance targeting most especially plasma membranes and macromolecules depending on the cell type and degree of exposure (Joye et al., 2004; Aylan and Metin, 2015). The antioxidant defense is made up of enzymes like superoxide dismutase, peroxidases lipases, and proteases as well as non-enzymatic systems including glutathione, thiols, and vitamins C and E (Niki, 1997). These antioxidants can be endogenous as well as exogenous and work as preventive, repair, and radical scavenging systems for defense from oxidative stress (Joye et al., 2004). In the right concentrations, ROS are useful in tissue repairs, angiogenesis, and cell proliferation (Yasuda et al., 1999) but when they are in high concentrations, they cause apoptosis and cell death, mutagenesis, carcinogenesis, and mitochondrial dysfunction (Juan et al., 2021). On the other hand, reactive nitrogen species (RNS) are molecules, for example, nitric oxides (NO), nitrogen dioxide (NO_2_), and peroxynitrite (ONOO^.^) (Klebanoff, 1980). Nitric oxide (NO^.^) is synthesized from l-arginine and molecular oxygen (O_2_). This is catalyzed by the nitric oxide synthase enzyme. On the other hand, NO^.^ and O_2_^.-^ react spontaneously and rapidly to produce peroxynitrite (ONOO^−^) which can be detoxified by isomerization to nitrate (NO_3_^−^) (Lugrin et al., 2014).

Given their highly reactive nature which is ascribable to the availability of valence shell electrons existing in an unpaired form or non-static bonds, and the inability of the antioxidant repair and preventive mechanisms to balance these ROS, they are able to cause cell and tissue damage (oxidative stress). This happens when they react with macromolecules such as proteins, nucleic acids, and carbohydrates by impacting various signaling pathways like the mitogen-activated protein kinase (MAPKs), Keap-1-Nrf2-ARE, and PI3K-Akt (Nayansi et al., 2017). These reactions often take place during environmental stress from radiation, diet, lifestyle, and exposure to heat molecules (Ray et al., 2012). A consequence of such reactions is their implication in accelerated aging, and pathologies such as autoimmune diseases, cardiovascular diseases, fibrotic diseases, infectious diseases, obesity, and cancers (Brieger et al., 2012).

Due to some of these pathogenic diseases which are associated with oxidative stress, the release of inflammatory stimuli, and signals such as peroxiredoxin 2 (PRDX2) (Salzano et al., 2014), the inflammatory process is triggered as a defense mechanism. This results in the pro and anti-inflammatory cytokines emission like the Nuclear Factor-kappa B/active protein-1 (NF-*κ*B/AP-1) and tumor necrosis factor-alpha (TNF-*α*) (Hussain et al., 2016). Inflammation is the body’s natural defense against injury, pathogens, and diseases from both endogenous and exogenous sources (Pahwa et al., 2022). It can be acute when short-lived or chronic when long-term and marked with inflamed cell continuous infiltration by white blood cells such as macrophages and lymphocytes. This is a consequence of the body being on constant alert giving rise to the production of inflammatory cytokines and enzymes causing cell damage. As a result, there is a development of numerous disease conditions like cardiovascular diseases, fibrosis, arthritis, and cancers which are the major causes of death worldwide (Khansari et al., 2009; Needham et al., 2019; Pahwa et al., 2022). The course of inflammation is characterized by the emancipation of cytokines, for example, TNF-α, IL-6, and RONS by NADPH oxidase found on neutrophil surfaces in a process called oxidative burst (Sharma et al., 2019) and hence depletion of antioxidants (Ryan et al., 2004; Khansari et al., 2009). Inflammation leads to the continuous production of ROS from inflammatory cells that overwhelm cellular antioxidants and can cause genomic mutations and extensive DNA damage (Coussens and Werb, 2002). To target the deleterious effects of inflammation and oxidative stress, regulation at the transcriptional and posttranscriptional levels has been targeted in recent years as they present a vast array of possibilities for gene expression control through the action of non-coding RNAs (ncRNAs).

Non-coding RNAs (ncRNAs), once referred to as dark matter, are RNA genes transcribed from DNA but not translated to proteins (Moreira et al., 2008). They are made up of housekeeping ncRNAs such as transfer RNA (tRNA) of 76–90 nucleotides (nt) in length, ribosomal RNA (rRNA) 120 to 4,500 nt, small nuclear RNA (snRNA) 100–300 nt, small nucleolar RNA (snoRNA) 60 to 400 nt, telomerase RNA (TERC), tRNA-Derived Fragments (tRF) 16–28 nt and tRNA halves (tiRNA) 16–28 nt (Zhang and Niu, 2019). These housekeeping ncRNAs are highly abundant in cells due to their functions which range from translation, pre-mRNA splicing, trans-translation and telomeric DNA synthesis (Lodish et al., 2000; Uchida and Adams, 2019). On the other hand, regulatory ncRNA production is dependent on the stage of the organism’s development and cell differentiation. In addition, production is equally dependent on external stimuli. They include microRNA (mRNA) with lengths from 19 to 23 nt, piwi-interacting RNA (piRNA) 26–31 nt, circular RNA (circRNA) 100–10,000 nt, small interfering RNA (siRNA), enhancer RNA (eRNA) 50–2,000 nt (Gusic and Prokisch., 2020). These riboregulators as they are sometimes called (Erdmann et al., 2001) function mostly in transcriptional and posttranscriptional regulation of gene expression (Szyman’ski and Barciszewski, 2002). Generally, all regulatory ncRNAs with length <200 nt are referred to as short ncRNAs with miRNA being the most abundant (Lagos-Quintana et al., 2001) while >200 nt are long ncRNAs (lncRNAs) which are the most abundant RNAs. lncRNAs have characteristics similar to mRNAs such as the presence of polyadenylated tail, methylated cap at 5′ end, and presence of introns and exons (Gusic and Prokisch, 2020), however, their level of expression is low and depends on a number of factors such as the physiological state, pathological state and tissue-dependent expression (Kornienko et al., 2016; Fazal et al., 2019). lncRNAs interact with nucleic acids to control gene expression at the level of the cytoplasm and nucleus giving them diverse functions such as enhancers, decoys, scaffolds, sponges (Charles et al., 2018) and more complexity (Esteller, 2011; Statello et al., 2021). ncRNAs regulate gene expression through histone modifications, DNA binding and recruitment of chromatin modifiers, heterochromatin formation, and gene silencing (Latronico and Silveira, 2018) and their differential expression has been observed in a variety of disorders. Owing to these outstanding features of ncRNAs, they present themselves as a propitious area that is being targeted for diagnostic and therapeutic purposes.

Natural products (NP) and their derivatives have been used for various disorders like diabetes, cancers, obesity, neurological disorders, and so on (Abdullah, 2020). They are organic compounds produced by living organisms such as insects, plants, animals, humans, marine, and microorganisms from secondary metabolism (Lippincott and Wilkins, 2020). They are classified into primary and secondary metabolites having antibacterial, antioxidant, antiviral, and anti-inflammatory properties (Dutta et al., 2019) and are used in both traditional and modern medicine for disease treatment. They are relatively low in cost, readily available, easy to apply, and of lesser side effects, (Dutta et al., 2019). Although usage of natural products is limited by extinction and/or inaccessibility of some species from which they are derived, complex and time-consuming isolation process and high complexity and instability of compounds found in NP (Enna et al., 2001; Huang and Hu, 2021), they are still advantageous in their relatively low costs, availability, easy application and of lesser side effects, (Dutta et al., 2019). Natural products have the ability to penetrate just as easily and reach their targets within or on the cell. Thanks to their diversity, NP possess new and innovative strategies that can aid in novel drug discovery (Dzobo, 2022). These have attracted researchers more towards their exploitation in disease therapy thereby bringing us to the purpose of this review which is aimed at highlighting some of the natural products that modulate the expression of ncRNAs involved in oxidative stress and inflammation-related disorders.

## 2 Disorders associated with oxidative stress and inflammatory imbalance

### 2.1 Disorders associated with oxidative stress

Oxidative stress is an abnormal state that the cells or tissues of an organism sometimes go through when they are subjected to endogenous or exogenous production of radical (or reactive) oxygen species that exceed their oxidizing capacity (Migdal and Serres, 2011).

Depending on the excessive production of oxygen radical species or a decrease in antioxidant capacities, oxidative stress can, through genomic, metabolic, and functional modifications, induce the development of different pathologies (Roy et al., 2017). It can therefore be a secondary element involved in the establishment of a disease or an element participating in the complications of immune diseases. It is not only implicated in the progression of several metabolic diseases such as type 2 diabetes (Giacco and Brownlee, 2010) and gestational diabetes mellitus (Lopez-Mejia and Fajas, 2014), but also in the development of age-related diseases, for example, atherosclerosis (Migdal and Serres, 2011), cancer (Kamal et al., 2022), cataracts, muscle degeneration, neurodegenerative diseases (such as Alzheimer), corneal dystrophy (Eddaikra and Eddaikra, 2021). Also, oxidative stress is implicated in heart failure, hypertension, infertility (Ménézo et al., 2012), and neuromuscular disorders (Mezdour et al., 2017).

In addition, it has been demonstrated that in most cases, oxidative stress is the cause of chronic inflammatory reactions, thus promoting the development of several diseases and *vice versa*.

### 2.2 Disorders associated with inflammatory imbalance

Inflammation is the body’s natural defense when in contact with harmful stimuli such as pathogens, irritants, and damaging cells. This mechanism initiates the healing process (Aghasafari et al., 2019) and is found in various metabolic diseases (Rémy et al., 2022), microbial, and genetic diseases, amongst others. The overexpression of this mechanism through the excessive production of inflammatory mediators can be at the origin or partake in the onset and progression of various diseases.

The presence of chronic inflammation contributes to the progression of certain diseases like type 2 diabetes (Donath, 2021), cancers, cardiovascular diseases, atherosclerosis (Castellon and Bogdanova, 2016), the pathogenesis of hypertension (Liang X. et al., 2020), and inflammatory bowel diseases (for example, Crohn’s disease and recto -hemorrhagic colitis) (Calle and Fernandez, 2012). In certain pathologies such as renal failure where patients are constantly on dialysis, oxidative stress is coupled with chronic inflammation, making the healing process of these dialysis patients even more complicated (Belaïch and Boujraf, 2016).

### 2.3 Relationship between inflammation and oxidative stress

Oxidative stress and inflammation are strongly related and associated with major diseases such as obesity, diabetes, cardiovascular and neurodegenerative diseases, so much so, that if one is the major event in a disorder, the other will come later as a consequence and accentuate the former. Oxidation of macromolecules like proteins and lipids during oxidative stress cause modifications which trigger innate immunity by acting as damage-associated molecular patterns (DAMPs) and pathogen Associated Molecular patterns (PAMPs) which bind to Pattern Recognition Receptors (PRR) like toll-like receptor (TLR) that lead to signal transduction thereby activating factors of transcription like NF-*κ*B and trigger inflammatory signals by inducing gene expression and recruiting immune cells (Tabas and Glass, 2013; Lugrin et al., 2014).

Furthermore, TLR has also been found to cause oxidative stress through the unbalanced production of proinflammatory and anti-inflammatory cytokines (Lavieri et al., 2014). Marseglia et al. demonstrated that levels of TNF- α are deregulated in weight loss and obesity, influencing the immune system, and the inflammatory process and inducing oxidative stress. OS is achieved through the production of ROS when TNF-α binds specifically to some receptors thereby promoting the NF-κB signaling pathway (Wang and Trayhurn, 2006; Marseglia et al., 2014). The latter equally states that the accumulation of adipose tissues also favors the synthesis of cytokines like TNF-α, IL-1, and IL-6 leading to the increased assembly of RONS by monocytes and macrophages which cannot be balanced by antioxidants due to their depletion in obese people thereby promoting oxidative stress. Inflammation from NF-κB pathway activation has also been found to be triggered by ROS-induced DNA-base modifications. In this situation, oxidative stress renders the redox potential oxidized at the level of cysteine and disulfide cysteine in plasma. This further causes the linkage of monocytes to endothelial cells thereby activating NF-*κ*B and causing inflammation (Iyer et al., 2009).

Moreover, inflammation is able to trigger oxidative stress through the release of enzymes, reactive species, and chemical mediators such as cytokines and nitric oxide at inflammatory sites (Subrata, 2016). In lung cancer, for example, increased expression of NADPH oxidase 4 (NOX4) by IL-6 which is an inflammatory cytokine induces ROS production (Li et al., 2015). Yongzhong et al. in their experiment were able to demonstrate that the production of H_2_O_2_ which was dependent on activation of both the Stat1-and NF-κB pathways by human pancreatic cancer cell lines was enhanced by the IFN-γ and LPS pro-inflammatory stimulated transcription of Duox2 and DuoxA2 (Yongzhong et al., 2013). Furthermore, the development of dysplasia has been linked to the production of ROS resulting from inflammatory infiltration. The production of ROS and inflammation have equally triggered the development of benign polyps into colorectal cancers when they impair pathways such as the Wnt/-catenin and/or base excision repair pathway (Zhao et al., 2022). Hence it is important to look at the course of oxidation in pathology with inflammation as the primary pathophysiological process and *vice versa* as these two turn out to be related. This offers a better chance to discover the cause of the pathology and increases the chances of better treatment or management.

## 3 Non-coding RNAs

### 3.1 Classification of non-coding RNAs

Non-coding RNAs (ncRNAs) are non-protein coding genes that can either be regulatory or housekeeping based on their functions. Regulatory ncRNAs have epigenetic functions, transcriptional, and post-transcriptional regulatory functions as well. Several types of ncRNAs exist ranging from snoRNA, snRNA, tRNA, rRNA, lncRNAs, and short ncRNAs like miRNAs, siRNAs, and piRNAs.

### 3.2 Non-coding RNAs involved in oxidative stress and inflammation-related disorders

Oxidative stress induces exaggerated inflammatory reactions and damage to lipids, proteins, and nucleic acids or causes damage to certain tissues that can lead to complications. Several studies suggest that non-coding RNAs may function as key modulators in the response to oxidative stress and inflammation associated with pathological states (Ghafouri-Fard et al., 2020) with a good number of them investigated with natural products as shown on Table 1.

**TABLE 1:** Non-coding RNAs modulated by Natural Products in Oxidative Stress and Inflammation Related Disorders

Disorder	Non-coding RNA modulated by natural products	References
Cancers (lncRNAs)	GAS5, HOTAIR, H19, RPI-179N6.3, MUDENG, AK056098, AK294004, AF086415, AK095147, HIF-1α, FOXM1, PCGEM1, AT102202, HOTAIR, DBH-AS1, PCGEM1, PRNCR1, PCAT29, AK001796, MALAT1, u-Eleanor, LINC00978, CCAT1, lncRNA BDLNR, NKILA, PAX8-AS1-N, CASC2, MEG-3, GAS-5, MHRT, NEAT1, WDR7-7, EWSAT1, lncRNA00364, MIR210HG, CFLAR-AS1, UBL7-AS1, MIR210HG, PAX8-AS1-N, WDR7-7, EWSAT1, lncRNA-PVT1, linc-PINT, lncRNA-ROR, XIST, Tusc7, lncRNA-NBR2, lncRNA-UCA1, SOX2OT V7, LINC00511, SPRY4-IT1, lncRNA-TTTY18, C3orf67-AS1, RFX3-AS1, STXBP5-AS1, BANCR, TUG1, PCAT29, ZFAS1, LINC01116, Loc344887, lncRNA circ-PLEKHM3, STAT5A, STAT3, PTTG3P, BISPR, CRNDE, PCAT1, PVT1, SNHG16, SNHG7, ZRANB2-AS2, CDKN2B-AS1, ZFAS1, FLJ36000, ST70T1, MRAK052686, MIR155HG, GUCY2GP, LINC00623, H2BFXP, PANDAR, RP1-179N16.3, ZRANB2-AS2, DIO3OS, AK001796, ANRIL, LINC01121, HULC, TERRA, LINC00261	Yan et al. (2015), Al Aameri et al. (2017), Beaver et al. (2017),, Palmieri et al. (2017), Saghafi et al. (2019), Chen et al. (2021a), Chen et al. (2021b), Irshad and Husain (2021), Kalhori et al. (2021), Ma et al. (2021), Mishra et al. (2021), Ruiz-Manriquez et al. (2021), Cesmeli et al. (2022), Giordo et al. (2022), Sadegh et al. (2023)
Cancers (circRNAs)	circ-PRKCA, circ-ZNF83, circ-PLEKHM3, circ-FNDC3B, circ-102115, circSATB2 and circFOXM1	Li et al. (2022), Xiaoqing et al. (2021), Ji-An et al. (2021), Sifan and Fang (2021), Li et al. (2021), Bowen and Joong (2016), Daoqi et al. (2020), Jiabin et al. (2019), Zhang R. et al. (2021)
Cancers (miRNA)	miR-122-5p, miR-34a, miR-424, miR-503, miR-125b-5p, miR-200c-3p, miR-409-3p, miR-122-5p, miR-542-3p, miR-17 family, miR-96, miR-221, miR-520h, miR-16, miR-210, miR-7–1, miR-99a, and miR-21a, miR-98-5p, miR-92, miR-93, miR-106b, miR-141, miR-143 miR-663, miR-200c, miR-25, miR-92a-2, miR-103–2, miR-103–1, miR-222, miR-let 7, miR-27a, miR-125b, miR-21, miR-200b, miR-200c, miR-let-7c, miR-let 7b, miR-let 7d, miR- 146a, miR-140, miR-29a, miR-155, miR-27a, miR-19a, miR-19b, miR-32-5p, miR-214-3p, miR-134, miR-512-5p, miR-21-3p, miR-21-5p, miR-130a, miR-27a, miR-491, miR-141, miR-101, miR-429, miR-409-3p, miR-20a, miR-34c, miR-145, miR-31, miR-137, miR-221/222, miR-126, miR-15a, miR-186, miR-451a, miR-370, miR-373, miR-526b, miR-375, miR- 487b, miR-7f-1, miR-9, miR-203, miR-328, miR-1, miR-3p, miR-1260b, miR-1290, miR-196b, miR-124, miR-30e, miR-10a, miR-663, miR-744, miR-126-3p, miR-720, miR-1280, miR-128, miR-453, miR-494, miR-let-7a, miR-let-7e, miR-let-7f, miR-522-3p	Ahmad et al. (2012), Parasramka et al. (2013), Yu et al. (2013), Shakir et al. (2014), Dhar et al. (2015), Sanjeev et al. (2015), Venkatadri et al. (2016), Wu and Cui (2017), Abbasi et al. (2018), Geng et al. (2018), Chen et al. (2021a), Chen et al. (2021b), Ma et al. (2021), Ruiz-Manriquez et al. (2021), Yao et al. (2021), Javaid et al. (2022)
Sepsis	MALAT 1	Wang W. et al. (2021)
Diabetic nephropathy	miR-18a-5p, miR-33, miR-122, miR-363-3p	Xu et al. (2017), Shu et al. (2020)
Obesity	miR-155, miR-539-5p	Gracia et al. (2016), Eseberri et al. (2017)
Asthma	miR-34a	Alharis et al. (2018)
Liver Fibrosis	miR-20a	Zhu et al. (2020)
Multiple Sclerosis	miR-124	Gandy et al. (2019)
Rheumatoid arthritis	Linc00052, MALAT 1, miR-126-5p	Mishra et al. (2019)
Cardiovascular diseases	miR-29/B-2–5, miR-29, miR-181b, miR-21, miR-663, miR-30c2, miR-155, miR-34a, miR-21, miR-20b, miR-27a, miR-9, miR-21, miR-638, miR-150-5p, miR-204, miR-663, miR-149, miR-133, lncRNA sONE, MALAT 1	Bladé et al. (2013), Mishra et al. (2019), Ren et al. (2020), Ramanujam et al. (2021), Elham et al. (2022)
Inflammatory Bowel Disease (IBD)	miR-124, miR-125b, miR-31, miR-126, miR-155, miR-214, miR-369-3p, miR-191a, miR-132, miR-145, miR-17, miR-146, miR-16–1, miR-663, miR-21	Zhang R. et al. (2021)

#### 3.2.1 Short non-coding RNAs

Studies have shown various circular RNAs (circRNAs) that are regulated by oxidative stress and mediate ROS production as well as promote ROS-induced cell death, cell apoptosis, and inflammation (Li Y et al., 2020; Liang J et al., 2020). Some circRNAs implicated in oxidative stress and inflammation-related disorders are circ-PRKCA, circ-ZNF83, circ-PLEKHM3, circ-FNDC3B, circ-102115 circSATB2 and circFOXM1 on which natural products modulation have been investigated in cancers and other disorders (Jiabin et al., 2019; Ji-An et al., 2021; Sifan and Fang, 2021; Xiaoqing et al., 2021; Zhang W. et al., 2022).

MicroRNAs (miRNA); with lengths between 20 and 24bp are single-stranded ncRNA with diverse functions ranging from activation or suppression of gene expression and also the control of transcription and translation. Several reviews have outlined the role of miRNAs in oxidative stress and inflammation-related disorders such as Tahamtan et al. (2018); Konovalova et al. (2019); Infante-Menéndez et al. (2023). Recent study aimed at identifying RNAs involved in the regulatory processes in various pathologies related to inflammatory disorders reveal miR-20b, miR-149, miR- 21, miR-150-5p, miR-663 involvement in cardiovascular diseases, miR-126-5p in rheumatoid arthritis, miR-146, miR-124, miR-16–1, miR-132, miR-145 in inflammatory bowel disease (Zhang S. et al., 2021). Furthermore, miR-20a is involved in the modulation of inflammation in fibrosis-related diseases (Zhou et al., 2020). In the case of cancer, numerous miRNAs have been highlighted to be involved in different types of cancers (Table 1) such as miR-122-5p, miR-200 family, miR-205, miR-145 in breast cancers, miRNA promoter (such as miR-137, miR-9, miR-let-7) hypermethylation enhances the development of colorectal cancer (Venkatadri et al., 2016).

#### 3.2.2 Long non-coding RNAs

A lot of research has been carried out so far on long non-coding RNAs (lncRNAs) involved in oxidative stress and inflammation-related disorders with some already existing reviews such as those of Wang Y. et al. (2019); Soudeh et al. (2020); Kalhori et al. (2021); Xu and Zhang (2022); as well as Zhao et al. (2021) which outline lncRNAs with some of their mechanisms of action in oxidative stress and inflammation-related disorders. This section reviewed a few which have been investigated with natural products and expressed in disorders such as diabetes, cancers, autoimmune diseases, and neurodegenerative diseases (Parkinson’s diseases and Alzheimer’s diseases) with more equally found in Table 1.

MALAT-1 is involved in oxidative damage and complications related to diabetes such as diabetic nephropathy, vascular complications and diabetic retinopathy, and diabetic cataract. As its name suggests, it has been demonstrated that MALAT-1has an oncogenic role in various cancers, where it has been found in upregulated levels in the renal cortexes of C57BL/6 mice with streptozocin-induced type 1 diabetes (STZ) (Hu et al., 2017). The work of Hu et al. revealed that there is a mechanism of retroactive regulation between MALAT-1 and beta-Catherine related to podocyte damage caused by high amounts of glucose (Hu et al., 2017). In addition, the silencing of MALAT-1 induces a fall in the regulation of OS and inflammatory reactions in the kidney of diabetic mice. Furthermore, MALAT-1has a negative regulatory action on miR let-7f and increases *the krüppel-type factor 5* (KLF5) (Chu et al., 2022). *Krüppel-like factor* 5 is able to bind directly to the NADPH oxidase promoter4, which induces high expression of NADPH oxidase (NOX)4 in human AC16 cardiomyocyte cells thus contributing to oxidative stress (Zhang H. et al., 2021). Therefore, MALAT1 could cause OS in diabetic kidney tissue by KLF5/NOX4 signal regulation. MALAT-1 is equally linked to diabetic retinopathy and diabetic cataract. Almost all patients with type I diabetes and 60% or more of type II diabetic patients suffer from diabetic retinopathy during their lifetime (Kyriazis et al., 2021). The role of MALAT-1 has been delineated in diabetic retinas through experimental investigation on clinical samples such as type I diabetes mice induced by streptozocin (Fong et al., 2014) and type II diabetes mice (Yan et al., 2014).

HOX antisense intergenic RNA (HOTAIR) plays a role in oxidative stress in high glucose-induced human mesangial cells (HMC) through miR-147a (Wang et al., 2022). This lncRNA could be linked to oxidative stress and inflammation associated with neurodegenerative diseases such as Alzheimer’s and Parkinson’s Diseases. It has been also reported as a key negative modulator for oxidative stress in the myocardial ischemia-reperfusion (I/R) injury (Meng et al., 2020). Rheumatoid arthritis is another inflammatory disease in which HOTAIR has been found to be upregulated leading to increased enrolment of macrophages or by acting as a sponge on miR-126-5p. In sepsis, it has been found to enhance the excretion of inflammatory cytokines IL-1β, IL-6, and TNF-α, thereby reinforcing inflammation (Zhang J. et al., 2020).

Gene 3 expressed by the mother (MEG3), also known as gene trap locus 2 (Gtl2), is located on chromosome 14q32.3 in locus *Dlk1-Dio3* (Chen et al., 2021a; Chen et al., 2021b). Evidence has shown that MEG3 interacts with several microRNAs involved in the regulation of oxidative stress, such as miR181a and miR-145 (Shenouda et al., 2011; Deng et al., 2020). Its involvement has also been reviewed by Hong et al. in bone diseases and osteogenic differentiation (Hong et al., 2020).

Growth arrest-specific 5 (GAS5) enhances elevated glucose-induced kidney damage by oxidative stress reduction and can serve as a miR-221 sponge *via* direct and agonote2-dependent targeting (Ge et al., 2019). Its sponging abilities equally link them to miR-223-3p, to achieve their effect on inflammatory responses in the microglia cells in Parkinson’s disease (Wei et al., 2020). In addition, miR-221 is able to induce the downregulation of proteins related to proliferation and fibrosis by targeting sirtuin1 (SIRT1) which is a deacetylase protein that helps counteract oxidative stress in various diseases, including diabetes (Baldinu et al., 2004; Meng et al., 2020). In high glucose-induced human tubular cells, GAS has been proven through its heightened expression to be an inhibitor of the inflammatory and oxidative stress process by targeting miR-452-5p (Xie L. et al., 2019). The same anti-inflammatory effect on lipopolysaccharide-induced inflammation is seen in sepsis through GAS 5 targeting of miRNA-23a-3p (Zhenping and Dan, 2021).

NEAT 1 has an oncogenic role in various cancers. It is involved in the regulation of many other oxidative stress- and inflammation-related diseases (Chen et al., 2019; Cui-Cui and Fang., 2019). Its effect on inflammation is achieved through the NF- κB signaling pathway. NEAT enhances oxidative stress by inhibiting antioxidant enzymes such as SOD.

Other ncRNAs involved in oxidative stress and inflammation-related disorders are circular RNAs such as circ-PRKCA, circ-ZNF83, circ-PLEKHM3, circ-FNDC3B, circ-102115 circSATB2 and circFOXM1 on which natural products modulation have been investigated on in cancers and other chronic diseases (Zhang H. et al., 2022).

Much work has been done on ncRNA regulation by natural products in cancers, however, the effect of natural products on ncRNAs such as lncRNAs and circRNAs in other oxidative stress and inflammation-related disorders including neurodegenerative diseases, diabetes, obesity, liver and cardiovascular diseases still warrant a lot of research. It is also important to explore these aspects of research more on human subjects.

## 4 Natural products involved in oxidative stress and inflammation-related disorders

Natural products have different classifications, one of them being the broad classification based on the structural features and biosynthetic origin into alkaloids, polyphenols, phenylpropanoids (which are phenylalanine derived), polyketides (from malonate and acetate), and terpenoids (from isoprene, glycosides) (Osbourn and Lanzotti, 2009; Phurpa, 2018) (see Table 2 below). The four main sources from which natural products are obtained include; animals, plants, marine organisms, and microorganisms (Jabeen et al., 2014), and have a wide variety of uses in therapeutics. About 80%–90% of the world’s population is dependent on traditional medicine derived from natural products for primary healthcare and about 73% of pharmaceutical drugs or products have been derived from them (Phurpa, 2018). These products carry antioxidant, anti-inflammatory, anti-cancerous, and immunomodulatory potentials which could be exploited for the development of novel therapeutic strategies.

**TABLE 2:** Natural products, subtypes, and their therapeutic uses.

Natural products	Sub products and origins	Therapeutic activity	References
Alkaloids	Nitrogen containing compounds with an indole ring. Isolated from plants, bacteria, fungi and animals	Anaesthetic, antioxidant, anxiolytic, antimalaria (quinine), anticancer (homoharringtonine), cardioprotective, anti-inflammatory, analgesic (morphine), antiasthma (epinephrine), stimulant (nicotine)	Chaves et al. (2016), Kurek (2019)
Polyphenols	- Non-flavonoids: Cucumin, from the *turmeric longa plant* resveratrol from plants like (grape, pomegranates, blackberries), lignans, phenolic acids - Flavonoids: Plant-based foods (rich in flavones, flavonols, isoflavones, catechins, wogonine, *etc.*) such as fruits, dark vegetables, dark chocolate, tea, red wine, cereals. They are composed of 15 carbon atoms, 2 phenyl rings and 2 heterocyclic rings	Anti-inflammatory, anticancer, immunomodulatory effects, antioxidant, immunorestorative	Miranda et al. (1991), Chen et al. (2004), Veronique and Christine (2012), Singla et al. (2019)
Phenylpropanoids	Sourced from fruits, vegetables, cereal plants, beverages, herbs	Antioxidant, antidiabetic, anticancer, anti-inflammatory, antimicrobials. Examples include eugenol and cinnamaldehyde	Sharma et al. (2016), Khatkar and Sharma (2019)
Polyketides	Gotten from several organisms including filamentous fungi. Synthesized by polyketide synthase and similar to fatty acids. Some examples include lovastatin from *Aspergillus terreus, Monascus ruber,* T. virens and T. atroviride	Immunosuppressants, anticancer, antibiotics. For example, lovastatin for hypercholesterolemia treatment, emodine as an anti-inflammatory and anticancer, chrysophanol as anticancer, antiulcer and hepatoprotective	Smith and Tsai. (2007), Ahmed (2014), Domitrović and Potočnjak (2016), Xie C. et al. (2019)
Glycosides	Isolated from plants (for example, *digitalus purpurea, helleborus, cotyledon*) Observed in amphibians (*Bufonidea*) and snakes (*Rhabdophis tigrinus*), There are several types when subdivided based on the nature of aglycone. For example,; coumarin, athraquinone, cyanogens, flavonoids, phenols, saponins	Treatment of heart failure and atrial arrhythmias Aninflammatory, analgesics, antiinflammatory, cardiotonic, antibacterial, antifungal, antiviral, and anticancer effects	Soto-Blanco (2022)

### 4.1 Natural Product’s modulation of non-coding RNAs involved in oxidative stress and inflammation-associated disorders

Natural products and their secondary metabolites have been used for decades as medicines either in combination with other product(s) or as stand-alone medicine. Secondary plant metabolites or specialized metabolites are a group of natural products or toxins produced by the lifeforms and function in the survival and fecundity of the entity. Phytochemicals possess biological characteristics such as antioxidant, anti-inflammatory and anticancer properties and are responsible for promoting health and preventing disease (Kashyap et al., 2021). Based on their chemical structures, phytochemicals are grouped into the following; phenols and polyphenols (e.g., quercetin, luteolin, genistein, resveratrol, calycosine, daidzein, and genistein), nitrogen-containing alkaloids (e.g., berberine and sanguinarine), terpenes (e.g., betulin), and sulfur compounds (e.g., sulforaphane) (Yang et al., 2019) (Table 2). Several studies especially on cancers have highlighted natural products in the modulation of oxidative stress and inflammatory disorders through their action on ncRNAs (Saghafi et al., 2019; Irshad and Husain, 2021; Kalhori et al., 2021; Piergentili et al., 2022) but more research to unveil their in-depth mechanism of action is warranted as well as their investigation in other oxidative stress and inflammatory disorders. This review has been able to outline some of these natural products and present a brief summary in Table 3.

**TABLE 3:** A summary of Natural Products that Modulate Non-coding RNAs and its mediated effect in a given condition.

Natural product	Source	Action on disease or condition	Effect on ncRNA	ncRNA mediated effect	Reference
**Baicalein** 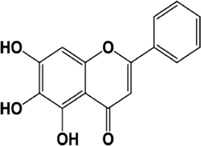	7-D-glucuronic-5,6-dihydroxy-flavone acid is a flavonoid compound extracted from the root of Scutellaria baicalensis	Anticancer effect on melanoma cancer cells (CI 50: 50 μM, 24 h)	Inhibition of CCAT1 lcRNA expression	inhibition of CCAT1 expression reduce cell invasion and migration by inhibiting the MEK/ERK and Wnt/β-catenin pathway axis by reducing the expression of the Wnt-3a, β-catenin, MEK and ERK genes	Yu et al. (2018)
		Anti-cancer: Hepatocellular carcinoma	Increase regulation of NKILA.	Increased NKILA expression reduces κBα phosphorylation inhibitors (IκBα) and nf-κB activity involved in the development of hepatocellular carcinoma. It also inhibits migration, proliferation and induces apoptosis	Wang et al. (2017)
**Tanshinone IIA** 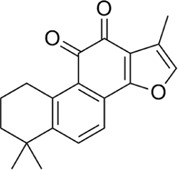	Phenanthro [1, 2-b] furan-10, 11-dione, 6, 7, 8, 9-tetrahydro-1, 6, 6-trimethyl) is a lipophilic diterpenoid extracted from the root of Salvia miltiorrhiza	Anti-inflammatory	Downregulation of the expression of miR-155 in colon cancer cell lines, miR-147, miR-184, miR-29b and miR-34c	LPS induced miR-155 over expression is modulated by Tan IIA through PU.1 regulation. This reduces the risk of colon cancer	(Tu et al., 2012; Fang et al., 2021)
		Atherosclerosis induced by *Porphyromonas gingivales*	Downregulation of miR-146b and miR-155	The downregulation of miR-146b and miR-155 significantly reduced the level of inflammatory factors such as CRP, OX-LDL, IL-1β, IL-6, IL-12, TNF-α, CCL-2, CD40 and MMP-2	Xuan et al. (2017)
**Geniposide** 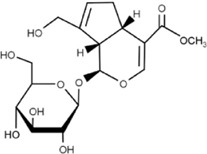	(GEN, methyl (1S,4aS,7aS)-7-(hydroxymethyl)-1 [(2S,3R,4S,5S,6R)-3,4,5-trihydroxy-6-(hydroxymethyl) oxan-2yL] oxy-1,4a,5,7a-tetrahydrocyclopenta [c]pyran-4-carboxylate) is derived from *Gardenia jasminoides* Ellis	Anti-inflammatory and cardiomyocytic	Upregulation of miR-145 expression	Inhibition of the pro-inflammatory factors IL-6, TNF-α and MCP-1, and suppression of the MEK/ERK pathway	Su et al. (2018)
**Carvacrol/Thymol** Carvacrol Thymol 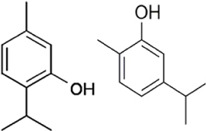	(5-isopropyl-2-methylphenol) and thymol (2-isopropyl-5-methylphenol) are isolated from the essential oil of *Origanum vulgare* L. and *Thymus vulgaris*	Anti-inflammatory in allergic asthma	Upregulation of miR-155, miR146a and miR-21	Upregulation of miR-155, miR146a and miR-21 leads to TLR 2, and TLR4 inhibition	Khosravi and Alder (2016)
**Triptolide** 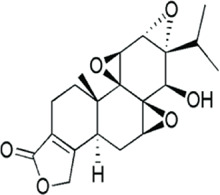	*Tripterygium wilfordii* is the thunder god vine from which triptolide is produced. Triptolide is an organic heterohepatacyclic compound	Diabetic nephropathy	Downregulation of miR-155-5p through BDNF upregulation and hence inflammatory injury and oxidative stress inhibition	miR-155-5p targets brain derived neutrophic factor (BDNF) which further reversed inhibitory action of miR-155-5p on triptolide protection on podocyte injury in mice. Genes COX-2 and IL-6	Gao et al. (2022)
**Oleacein** 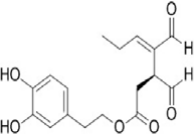	2-(3,4-dihydroxyphenyl) ethyl (4Z)-4-formyl-3-(2-oxoethyl) hex-4-enoate from *Olea lancea*	Anti-oxidant and anti-cancer	Interacting with miRNAs miR-193a-3p, miR-193a-5p, miR-34a-5p, miR-16-5p, miR-214-3p	Represses melanoma cell proliferation *via* interaction with microRNAs	Carpi et al. (2020)
**Curcumin** 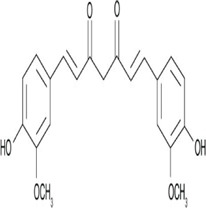	Natural polyphenolic compound extracted from turmeric longa	Anti-inflammatory, antioxidant and anti-cancer	Downregulation of UCA1 lncRNA	Inhibits the proliferation and promotes the apoptosis of cancer cells	Wang W. H. et al. (2018)
		Hepatocarcinoma	Inhibits the expression of lincROR	Blocks Wnt/beta catenin signalling pathway	Shao et al. (2020)
Resveratrol 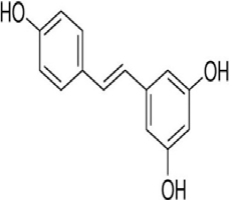	Resveratrol (3,5,4′-Trihydroxystilbene) is a polyphenolic phytoalexin derived from many plants such as grapes; Peanuts, pine, vines	Anti-cancer	Induction of tumor suppressors miR-34a, miR-424 and miR-503	Channel p53	Otsuka et al. (2018)
		Inflammatory, anti-inflammatory pathways	miR-155 and miR-663		Latruffe et al. (2015)
		Acute kidney injury induced by sepsis	Downward regulation of Malat1 and miR-205	Inhibition of the lncRNA MALAT1/miR-205 axis by resveratrol	Wang B. et al. (2021)
		Antiinflammatory	Increases miR-Let7A		Song et al. (2016)
		Allergy asthma and associated inflammation in the lungs	miR-34 downregulation	Overexpression of transcription factor FOXP3	Alharris et al. (2018)
		Toxicity induced by staphylococcal enterotoxin B	Downregulation of miR 193-a	Activation of the anti-inflammatory pathway	Alharris et al. (2018)
Allicin 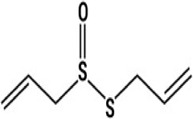	Also known as diallyl thiosulfinate Organic sulfur compound found in onion and other allium plants	Oxidation and autophagy in osteosarcoma	Upregulates MALAT1 expression	Regulation of MALAT1 miR-376a sponge expression *via* MALAT1-miR-376a-Wnt/B-Caterine signaling pathway	Xie et al. (2022)
Berberine 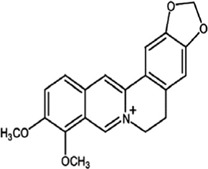	Berberine (5,6-dihydro-9,10-dimethoxybenzo [g]-1,3-benzodioxolo [5,6-a] quinolizinium) is extracted from the Chinese herb Coptis chinensis (Huanglian)	Antidiabetic	Modulation of miR-122, miR-30, lncRNA MRAK052686, lncRNA MRAK080926	AMPK and JNK port targeting	Chang (2017)
Omega 3 fatty acids 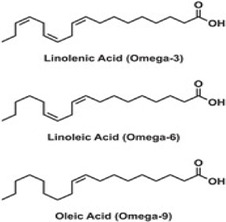	The main three are eicosapentanoic acid, docosahaenoic acid, alpha-linolenic acid Omega 3 fatty acids are obtained from fish, nuts, seeds, plant oils	Downregulation of phosphate and tensin homolog (PTEN) mRNA in hepatocytes and lead to onset of liver disorders	miR-21 upregulation	Inhibition of miR-21 target phosphatase and tensin homolog (PTEN)	Vinciguerra et al. (2009)

#### 4.1.1 Baicalein

Baicalein (5,6,7-trihydroxy flavone) is a flavonoid compound obtained from *Scutellaria species*. It has also been found to be present in the Indian trumpet flower. Baicalein has properties like neuroprotection, anti-inflammatory, and antioxidation. Baicalein has been shown to exert its anticancer effects in melanoma cancer cells by inhibiting the expression of CCAT1 (transcript 1 associated with colon cancer, its upregulation increases the melanoma index). Baicalein may reduce cell invasion and migration if inhibition of CCAT1 expression inhibits the MEK/ERK and Wnt/β-catenin pathway axis by reducing the expression of the Wnt-3a, β-catenin, MEK, and ERK genes, in addition to decreasing cell proliferation by increasing the percentage of apoptotic cells (Yu et al., 2018). Baicalein may reduce κBα phosphorylation inhibitors (IκBα) and nf-κ B activity by increasing the expression of NKILA interacting with nf-κ B, thereby inhibiting migration, proliferation, and inducing apoptosis. LNCRNA-NKILA is a negative regulator of NF-κ B activity and is downregulated in hepatocellular carcinoma (Wang et al., 2017). Wang L et al.’s experiment in 2017 on rat intestinal cells showed that pre-treatment with baicalein could reverse the effects of TNF-α-induced expression of miR-191a, which would improve the viability of rat small intestine epithelial cells (Pan et al., 2017).

#### 4.1.2 Tanshinone IIA

Tanshinone IIA is a lipophilic diterpenoid medicinal herb extracted from the roots of *Salvia miltiorrhiza* Bunge (Danshen). It is used in the treatment of diseases like diabetes, sepsis, arthritis, and cardiovascular diseases. Some research has been done on the effects of Tan II on cancers like colorectal cancer where they are able to exert their anti-inflammatory potential by reducing Hct116 and Ht29 cell proliferation and inhibiting microRNA-155 (Tu et al., 2012).

Furthermore, Tan IIA has been shown to reduce the level of expression of some cytokines, chemokines, and acute phase proteins, for instance, TLR4, MyD88, GM-CSF, sICAM-1, CXCl-1 and MIP-1α (Cheng et al., 2018). In addition, it significantly inhibits the mRNA expression levels of IL-1β, TNF-α, and COX-2, thereby suppressing lipopolysaccharide-induced activation (LPS) of the TLR4-NF-κ B pathway (Fan et al., 2016). Tan IIA has anti-inflammatory activity by exerting a negative regulation on the expression of miR-155, miR-147, miR-184, miR-29b, and miR-34c. Moreover, Tan IIA performs a protective function in atherosclerosis induced by *Porphyromonas gingivalis* by exerting a negative regulatory action on the expression levels of miR-155 thereby markedly reducing inflammatory factors like CRP, OX-LDL, IL-1β, IL-6, IL-12, TNF-α, CCL-2, and MMP-2 (Xuan et al., 2017).

#### 4.1.3 Geniposide

Geniposide is derived from *Gardenia jasminoides Ellis.* It is a medicinal iridoid glycoside compound with antidiabetic, anti-inflammatory and antioxidant, and cardioprotective properties. Studies performed on LPS-injured H9c2 cells have reported anti-inflammatory and cardiomyocytic protective effects of geniposide. It upregulates the expression of miR-145, inhibits the pro-inflammatory factors of IL-6, TNF-α, and MCP-1, and then suppresses the MEK/ERK pathway and at such inhibits apoptosis and promotes cell viability (Su et al., 2018). In diffuse large B-cell lymphoma, geniposide is able to knockdown lncRNA HCP5 thereby increasing miR-27b-3p (a target of HCP5) levels and so inhibiting cell proliferation and inducing apoptosis in this condition (Hu et al., 2020).

#### 4.1.4 Carvacrol/Thymol

Carvacrol or cymophenol and thymol (2-Isopropyl-5-methylphenol) are monoterpenoid phenol isomers found in thyme oil, pepperwort oil, and wild bergamot. These isomers have antioxidant, antihypertensive, anticancer, and anti-microbial properties. In chitin-induced models, Car/Thy had the potential to suppress inflammation allergic asthma by upregulating miRNAs and factors of inflammation such as; miR-155, miR146a, and miR-21, promoter of pro-inflammatory cytokines. More so SOCS, SHIP1, and miR-155 target and negatively regulate TLR-mediated inflammation and are inhibited by chitin. Abnormal expressions of TLR2, TLR4, SOCS1, SHIP1 and miR-155, miR-146a, and miR-21 can also be reversed by the combination of Car/Thy (Khosravi and Alder, 2016).

#### 4.1.5 Triptolide

Triptolide is derived from the medicinal herb *Tripterygium wilfordii*, (Yang Y. Q. et al., 2018; Yuan K et al., 2019; Tamgue et al., 2021). This triepoxyde diterpene has cytotoxic, anti-inflammatory, and anticancerous activities. Cytotoxic activities of triptolide have been highlighted in some tumors through induction of necrosis and apoptosis, and cell cycle arrest in several cell lines (Yang Y. Q. et al., 2018; Tamgue et al., 2021). The anti-inflammatory activities of triptolide are achieved through mechanisms ranging from the downregulation of NF-kb, p-TAK1, p-IκBα. It is able to reduce OS and inflammation in podocyte injury in diabetic nephropathy treatment through the downregulation of miR-155-5p which targets brain-derived neurotrophic factors. AP-1–controlled pro-inflammatory molecules such as TNF-α, IL-6, IL-12, IL-1β, and Ptgs-2 are also downregulated by triptolide in several cell types (Yang Y. Q. et al., 2018; Tamgue et al., 2021).

#### 4.1.6 Oleacein

Oleacein is an olive secoiridoid alcohol. It is known for its anti-inflammatory and antioxidant abilities which renders them a potential treatment option for neuroinflammatory disorders. Oleacein equally has anti-cancerous effects where it represses melanoma cell proliferation by interacting with several microRNAs like miR-193a-3p to target MCL1 and K-RAS, miR-193a-5p, miR-34a-5p, miR-16-5p, miR-214-3p and their mRNA targets encoding family proteins Bcl-2 and the mTOR pathway which opposes cell survival, proliferation, and apoptosis (Carpi et al., 2020).

#### 4.1.7 Curcumin

Curcumin is a polyphenolic compound extracted from *Curcuma longa* Linn*.* It has been demonstrated to have various properties such as anti-cancer, anti-inflammatory, antimutagenic, and antimicrobial, and is able to increase antioxidant production by the body (Mirzaei et al., 2018). The anticancerous effects of curcumin are portrayed by its ability to influence multiple signaling pathways like the NF-kB, MAPK, PTEN, and p53 which are all involved in the progression of cancers. For instance, curcumin inhibits progression and promotes cancer cell apoptosis by negatively modulating the lncRNA UCA1 (Wang R. et al., 2018), it also upregulates microRNAs such as miR-502c, miR-181b, miR-16, miR-15a, miR-146b-5p, and miR-132 while downregulating others like miR-19a, miR-19b (Piergentili et al., 2022). In hepatocarcinoma cells, it suppresses the expression of lincROR and blocks the Wnt/B-catenin signaling pathway activation (Shao et al., 2020) while another study demonstrates that it inhibits GAS5 expression in lung cancer by affecting major pathways such as NF-kB and STAT3 (De Bacco et al., 2011). Furthermore, curcumin is able through the miR-21/PTEN/Akt pathway, to reduce gastric cancer cell line proliferation by inducing apoptosis (Qiang et al., 2019). It is equally able to inhibit the expression of miR-21 and upregulate miR-34a expression in gastric cancer cells.

#### 4.1.8 Resveratrol

Resveratrol is a polyphenolic phytoalexin derived from many plants such as grapes; groundnut, pine, and vine which revealed anti-cancer effects (Rauf et al., 2018). There have been several research demonstrating the regulation of ncRNAs by resveratrol (Giordo et al., 2022). It inhibits breast cancer cell proliferation by inducing the expression of tumor suppressor miRNAs (miR-34a, miR-424, and miR-503) through the p53 pathway (Otsuka et al., 2018) and lncRNA LINC00978 (Deng et al., 2016). The link between resveratrol and lncRNAs correlation with inflammation and oxidative stress is still being studied and its mechanism of action is yet to be clearly understood. Resveratrol appears to have a modulatory effect on miRNAs, among which are miR-155 involved in the inflammatory pathways, miR-663; an anti-inflammatory ncRNA, and oncogenic miR-21 (Latruffe et al., 2015). In addition, Malat 1 lncRNA and miR-205 were downregulated in rats with resveratrol-treated sepsis-induced acute kidney injury compared to rats not receiving treatment (Wang B. et al., 2021), the same study suggests that inhibition of lncRNA MALAT1/miR-205 by resveratrol has a protective function in sepsis-induced acute kidney injury (Wang W. et al., 2021). Also, miR-Let7A decreases inflammation by upregulation after resveratrol treatment in human THP-1 macrophages (Song et al., 2016). The negative modulatory effect of resveratrol on miR-34 attenuates asthma allergy symptoms and associated inflammation in the lungs of mice *via* overexpression of the transcription factor FOXP3 (Alharris et al., 2018). Low regulation of miR-193-a by resveratrol has a protective action against toxicity induced by staphylococcal enterotoxin B by activation of anti-inflammatory pathways in mice (Alharris et al., 2018).

#### 4.1.9 Solarmargine

Solarmargine is a steroidal alkaloid glycoside derived from solanum plant species such as Solanum nigrum with anticancer activities (Kalalinia and Karimi-Sani, 2017). A study demonstrated that solarmagine regulates miR-155-induced apoptosis by repressing the expression of lncRNA HOXA1-AS and inhibits cancer cell proliferation (Meng et al., 2022).

#### 4.1.10 Allicin

Allicin or diallythiosulfinate is an organic sulphury compound found in onion and other allium plants such as garlic. It is extracted from the allium bulb and has anti-cancer, anti-inflammatory, antibacterial, and anti-viral effects (Ba et al., 2019). Physiologically, low doses of allicin have antioxidative effects in atherosclerosis through low-density lipid protein oxidation. It functions in the treatment of so many cancers like colorectal cancers, breast cancers, and lung cancers through its proapoptotic ability. Xie et al. were able to show that allicin was able to reduce MALAT1 expression and hence inhibited osteosarcoma proliferation and progression by targeting the Wnt/*β*-catenin pathway thereby regulating the genes in this pathway.

#### 4.1.11 Aqueous extract or pulp of Açai

Pulp of Acai is a source of polyphenols. Oxidative stress and inflammation make up some of the underlying conditions that lead to the advancement of non-alcoholic fatty liver disease. Acai pulp has been investigated in this condition and shown to prevent the oxidation of low-density lipoproteins (Pereira et al., 2016). *Flavon velutin* is an active ingredient found in acai and has been reported to act as an effective anti-inflammatory agent by blocking LPS-mediated production of TNF-α and interleukin-6 thereby inhibiting the activation of NF-κB and the MAPK pathway (Xie C et al., 2012). These two pathways are involved in the mechanism of action of certain LncRNAs such as NKILA (Liu B et al., 2015). Also, research has shown that acai can be useful against lung diseases. In this condition, it is able to exert its antioxidative effects through an inhibitory action on the NF-κB pathways and Nrf2/ARE pathway stimulation.

#### 4.1.12 Berberine

Berberine extracted from the Chinese herb *Coptis chinensis* (Huanglian), is an isoquinoline alkaloid that has been known to have antidiabetic effects by targeting pathways like the AMPK and JNK pathways thereby regulating ncRNAs. Some ncRNAs regulated by berberine include miR-122, miR-30, lncRNA MRAK052686, lncRNA MRAK080926 (Chang, 2017). By so doing, berberine is able to reduce stress in the endoplasmic reticulum and overcome insulin resistance (Chang, 2017). In type 2 diabetic patients, berberine has been known to help in weight gain reduction, reduction of blood lipid level production as well as antiglycaemic. Through hepatocyte nuclear factor-4α (HNF-4α) inhibition, miR-122 expression has been reported to decrease with the treatment of berberine. This promotes a fall in expression levels of enzymes like PEPCK and G6Pase involved in hepatic gluconeogenesis.

#### 4.1.13 Quercetin

Quercetin is a pentahydroxy flavone from the group of flavonoids of polyphenols. Plants containing this natural product include; green tea, red wine, and onions. It is commonly used but not limited to the treatment of heart conditions, diabetes, obesity, and certain cancers where they have been found to dysregulate miR-16, miR-145, MALAT-1, miR-155, miR-146a, miR-197, lncRNA SNHG7 and miR-223 (Piergentili et al., 2022). Studies have demonstrated that quercetin has a protective effect against free radicals from smoking and lowers lncRNA DBH-AS1 expression through antioxidant activities in hepatocellular carcinoma (Saghafi et al., 2019). Quercetin is also a core anti-inflammatory molecule with the ability to inhibit enzymes that are key regulators of the inflammatory process acting on its mediators through their influence on enzymes such as lipoxygenase and cyclooxygenase (Xiao et al., 2011). It also functions in lowering levels of LDL and increasing the dilation of major arteries which ease blood flow thereby preventing heart diseases.

#### 4.1.14 Genistein

Genistein (prunetol) is a tyrosine kinase inhibitor with a 4′,5,7-trihydroxyisoflavone chemical structure. Genistein is an isoflavonoid extracted from soybeans and fava beans. Genistein has been known for various biological and therapeutic properties for so many years. In tissue culture, for example, it is used as a phytoestrogen to induce cell differentiation. Studies have documented that genistein is able to reduce the severity of histopathological changes in acute pancreatitis through the action of its antioxidant, anti-inflammatory, and anti-apoptotic properties. (Heubach et al., 2015; Prasong et al., 2022)*.* Genistein has anti-cancerous properties as seen in its ability to suppress the progression of apoptotic cells in metastatic cancers and inhibit HOTAIR in renal and prostate cancer (Imai-Sumida et al., 2017). It also downregulates miR-34a in prostate cancer. It has also been widely researched in their modulation of ncRNAs in cancers like miR-151, miR-221, miR-222, miR-223 downregulation and miR-34a, miR-573-3p, miR-1296 upregulation in prostate cancers, miR-155 suppression in breast cancers, miR-34a, miR-200 upregulation in pancreatic cancer (Piergentili et al., 2022).

#### 4.1.15 Omega-3 fatty acids

Omega-3 fatty acids are healthy polyunsaturated fatty acids that exist primarily in food sources, especially oily fish. To date, there are several controversial effects of omega-3 fatty acids on oxidants and antioxidants. But that does not rule out their beneficial effects in lowering triglyceride levels and rendering protection against neurodegenerative and cardiac diseases through their anti-inflammatory, anti-allergic, and cardioprotective properties. Studies conducted by a randomized clinical trial (RCT) on patients reveal that omega-3 fatty acid supplementation significantly improves decreased malondialdehyde (MDA), total antioxidant capacity, and glutathione peroxidase activity. Thus, rendering omega-3 fatty acids as factors amplifying the antioxidant defense against ROS. (Javad et al., 2019). Omega- 3 fatty acids are also involved in liver diseases by targeting miR-21 which causes PTEN degradation (Vinciguerra et al., 2009). In cancers, omega-3 unsaturated fatty acids have also been found to regulate apoptosis-related miRNAs such as miR- 17, miR- 26a, miR-200a, miR-323 (Visioli et al., 2012).

The interaction of natural products with ncRNAs could be on the basis of several natural products modulating one ncRNA; for example, miR-21 regulation by sulforaphane, icariin, and piceatannol (Irshad and Husain, 2021) or one natural product having the potential of controlling several ncRNAs. The latter is seen in an example like curcumin which has the ability to modulate several lncRNAs such as H19, LINC00623, PVT1, PANDAR, AF086415 (Mishra et al., 2019). Future research can equally exploit the combined effects of some natural products on a particular ncRNA in order to see if they react antagonistically or synergistically in the moderation of a specific ncRNA of interest.

### 4.2 Natural products involved in oxidative stress and inflammation-related disorders with unknown effects on ncRNAs

#### 4.2.1 Zanthoxylum bungeanum

*Zanthoxylum bungeanum* Maxim (ZBM) is plant species of the Rutaceae family of traditional herbal medicine. It is widely found in Asia. The mature pericarp of ZBM is used for the treatment of diseases and as a spice Sichuan pepper used for cooking (Wang et al., 2020). It is a rich source of chemicals with about 140 chemical compounds being isolated from it including terpenoids, flavonoids, and alkaloids giving this plant a very vast array of therapeutic and medicinal functions. Antioxidant, anticancer, and hepatoprotective effects are found in Zanthoxylum alkylamides and comprises α-, β- and γ alkylamides (Ren et al., 2017). ZBM has been reported to attenuate liver injury by activating nuclear factor-κB (NF-κB) and mitogen-associated protein kinase (MAPK) signaling pathways. This is accomplished through the inhibitory action of phosphorylated p65/NF-κB, MAPK (including p38, JNK, and ERK1/2), and activation of transcription factor 3 protein expression, with further suppression of Bax, cytochrome c, caspase-9 and caspase-3 (Zhang et al., 2018). ZBM also has anti-inflammatory and anti-analgesic effects.

#### 4.2.2 Canna genus rhizome

It is a plant used in the traditional environment for its anti-inflammatory, analgesic and antipyretic properties. It is a rich source of flavonoids, phytosterols and phenolic acids. CGE has been reported to attenuate inflammation in ulcerative colitis by down-regulating LPS/TLR4 signal pathways, cleaving caspase-1, NLRP3 decreased protein expression, and NF-ҡB expressions (Mahmoud et al., 2021).

#### 4.2.3 Fuzi-ganjiang herb pair

Fuzi and ganjiang are widely used in China, Korea, and Japan in the treatment of ulcerative colitis (UC), vomiting, and heart failure for over 1,800 years (Huang et al., 2020). Fuzi-ganjiang contains a lot of alkaloids and has been outlined for its anti-inflammatory properties. Research carried out on DSS-induced mouse colon tissue by Huang et al. showed that 6-gingerol which is an active ingredient obtained after the combination of fuzi-ganjiang herb pair was able to inhibit the production of MPO and suppress the expression of inflammatory cytokines which in this case were IFN-γ, TNF-α, IL-1β, IL-6, IL-10, and IL-17A. the expression levels of inflammatory mediators like MPO, iNOS, and COX mRNA are also suppressed by this combination. Moreover, the MAPK, NF-κB, and STAT3 signaling pathways are also inhibited by this combination of Fuzi-gangiang in order to accomplish its anti-inflammatory activities (Huang et al., 2020)*.*

#### 4.2.4 Aronia melanocarpa

Generally used in the production of juices and jams. Another name for it is black chokeberry and has been found to have ethnopharmacological properties due to the richness in the biologically active molecules of the natural product with pharmaceutical and physiological effects (Li et al., 2022). In obesity-induced inflammation in adipose tissue, Aronia extract has been shown *in vivo* and *in vitro*, to inhibit phosphorylation of the NF-kB pathway and P65. Extracts from this plant have been found to possess hypolipidemic and antioxidative properties. For example, anthocyanin-rich fraction from this fruit plant was able to reduce the levels of ROS caused by high glucose through its scavenging action in pancreatic β cells. The fruit juice of black chokeberry has equally been shown to have hypoglycaemic functions (Valcheva-Kuzmanova et al., 2007).

#### 4.2.5 Peppermint

Peppermint (*Mentha piperita L*.) is extracted from the peppermint plant and contains important compounds such as menthol, iso-menthone, and neomenthol. It is used worldwide for its anti-inflammatory, antioxidant, antiviral, antimicrobial, and antibacterial activities among others (Sun et al., 2014). In inflammatory bowel disease, one of its key components menthols is able to decrease the production of inflammatory cytokines such as IL-1, IL-6, IL-23, and TNF- α and free radicals in colonic tissues. PEO is also able to enhance the upregulation of anti-inflammatory cytokines in irritable bowel syndrome (Zhao et al., 2022). However, indebt knowledge of the mechanisms of action in their various pharmacological effects is still to be uncovered. This is of importance due to the diverse use of these products universally giving reason to believe that it holds an affluent potential if investigated in its ability to target ncRNAs in oxidative stress and inflammation-related disorders.

#### 4.2.6 Gingerol

It is an amphiphilic phenolic lipid obtained from the ginger rhizome. Its anti-inflammatory activities have been documented in HDM-induced asthma in rats where 6-gingerol is able to cause a decline in neutrophilic inflammation by reducing levels of •NO, IL-6, TNF- α and myeloperoxidase (Babajide et al., 2022). It also has a protective role by decreasing proinflammatory cytokines and oxidative stress in liver diseases (Alsahli et al., 2021). Cote et al. (2022) in their review of the role of some phytochemicals in inflammation-associated disorders outlined gingerol activities in cancers where it was able to reduce inflammation thereby decreasing tumors, in cardiovascular diseases, and equally in fasting blood glucose, inflammation, and oxidative stress reduction in diabetes.

## 5 Non-coding RNAs involved in oxidative stress and inflammation-related disorders on which the effect of natural products has not been investigated

Several authors have documented the implication of natural product-modulated ncRNAs; especially miRNAs and lncRNAs in various types of cancers, still leaving much to be done with regard to other oxidative and inflammation-related disorders like neurodegenerative disorders (for example, Parkinson’s diseases and Alzheimer’s disease), atherosclerosis or rheumatic diseases with a good number of regulatory ncRNAs on which natural products have not been investigated (Gámez-Valero et al., 2020; Zhang W. et al., 2022; Infante-Menéndez et al., 2023). We, therefore, highlight in this section and in Table 4, some of these ncRNAs that have not yet been investigated with natural substances in the context of diseases related to oxidative stress and inflammation.

**TABLE 4:** Non-coding RNAs involved in Oxidative stress and Inflammation related Disorders on which the Effect of Natural Products has not been Investigated.

Non-coding RNAs	Oxidative stress and/or inflammatory disease	Target of the ncRNA	Action of ncRNA	Model	References
SnoRNA	Cancers -Non-small-cell lung carcinoma -Colorectal cancer -Breast cancer -Ovarian SNORD8	P53 Notch 1/c-Myc	Promote cell cycle arrest, induce apoptosis and inhibit cell proliferation by targeting its pathways. -Modulates the response of p53 and promotes cell proliferation. -Promotes cell proliferation when upgraded	Clinical cancer tissues and breast cancer cell lines	Zhang L. et al. (2020a), Zhang L. et al. (2020b), Cui et al. (2021), Huang et al. (2022)
siRNA	Pulmonary Fibrosis Cancers Human breast cancer Rheumatoid Arthritis	P53 Notch 1/c-Myc Bach 1 Notch 1 IL-1, IL-6, IL-18	Knockdown of Bach 1 (Bach 1 induces oxidative stress by targeting NrF2) -Down regulate Notch 1 expression hence increasing chemosensitivity -Increases chemoresistance through Notch 1 knowckdown siRNA targeted silencing led to downregulation of inflammatory and rheumatoid arthritis autoimmune components	Lungs of mice with bleomycin induced pulmonary fibrosis -Mice	Ye et al. (2012), He-da et al. (2015), Liu et al. (2017)
piwi RNA	Lung cancer -Liver cancer	mammalian target of rapamycin -piR-Hep 1 suppress cell death	-Inhibit cell migration and invasion by functional maintenance of phosphorylated erzin-radixin-moesin - piR-55490 suppresss cell proliferation through 3′ UTR binding to mammalian target of rapamycin -suppress cell death by activating signal transducer and activator of transcription 3 (Stat3)/Bcl-xL		Xi et al. (2020)
CircRNA 1. CircNCX1 2. CircACR 3. CircHIPK3	MiR-133a-3p Glycogenesis pathway miR-29a	Induces target 1 p53(CPIP1) Induces the decrease in glucose levels miR-29a sponge	Cardiomycytic cells SchwannRSC96 cell phone Endothelial cells	Apoptosis of cardiomycytic cells Alleviates SchwannRSC96 cell apoptosis excited by high glucose content, autophagy and ROS generation Protects endothelial cells from damage caused by oxidative stress and vascular dysfunctions *in vitro*	Li M. et al. (2018), Li Q. et al. (2018), Liu et al. (2019), Wang X. et al. (2019)
miRNAs 1. miR-210 2. miR-330a 3. MiR-145	Atherosclerosis Alzheimer’s Disease (AD) Diabetic cardiomyopathy	caspase VAV1, ERK1, JNK1, P38MAPK and Aβ Negative regulation of ARF6	An overexpression of miR-210 induces a Inhibition of apoptosis and a reduction in ROS and caspase levels in HUVEC treated with H2O2 Reduces mitochondria dysfunction and oxidative stress MiR-145 attenuated HG-induced inflammatory response and bone damage in cardiomyocytes by downregulating ARF6	Endothelial cells in the human umbilical vein (HUVEC) AD mice H9c2 cells	Banerjee et al. (2017), Zhou et al. (2018), Zheng et al. (2021)
SNHG16	Pneumonia	miR-146a-5p JNK and NF-κB pathways	promoting viability, restraining apoptosis and production of inflammatory cytokines	restoring Csingle bondC motif chemokine ligand 5 (CCL5) expression	Zhou et al. (2019)
MALAT 1	Diabetic kidney disease	KLF 5 downregulation	Inhibited MALAT1 suppressed podocyte injury, oxidative stress and inflammation in renal tissues of DN mice	Diabetes mice	Zhang X. L. et al. (2020)
MEG 3	Obesity Diabetes mellitus Diabetic nephropathy (DN)	Dynamin-related protein 1 (Drp1) miR-181 sponge	Impairs glucose homeostasis and increases insulin resistance MEG 3 knockdown relieves renal dysfunction, glomerular injury, promotes mitochondria fission, albuminuria Direct targeting in Ago2-dependent manner on miR-181 which targeted Egr-1, promoted MC fibrosis and inflammatory response	Diet induced mice Human podocytes and Meg3 podoyte-specific knockdown mice Mesanglial cells and DN rat models	Zha et al. (2019), Deng et al. (2020), Zhang X. L. et al. (2020)
GAS 5	Diabetic nephropathy (DN)	miR-221 sponge targeting of Sirtuin 1 matrix metalloproteinase 9 (MMP9)	sirtuin 1counteracts oxidative stress downregulation of MMP9 by EZH2 to alleviate inflammation and renal interstitial fibrosis in the kidneys	Mesanglial cells DN rats	Ge et al. (2019), Huang and Hu (2021)
lncRNA MIAT	Diabetic rettinopathy	Transforming growth factor (TGF)	Upregulates TGF- β1signaling	ARPE-19 cells	Li et al. (2020)
LINC00638	Rheumatoid arthritis	Inflammatory cytokines	Overexpression of LINC00638 activates Nrf2/HO-1 pathway and decreases IL-6, IL-17, IL-23, ROS levels	Peripheral blood mononuclear cells (PBMCs)	Yanqiu et al. (2022)
H19	Diabetes	miR-657	Reverses inflammation and oxidative stress *via* miR-657 targeted inhibition of voltage dependent eanion channel 1 (VDAC1)	Diabetic mouse	Sun et al. (2016)
LINC01619	Diabetic nephropathy	miR-27a	Oxidative stress and podocyte injury enhancement by sponging on miR-27a, which negatively targets FOXO1 and activates ER stress	HG podocytes and diabetic rats	Bai et al. (2018)

As part of the regulation of oxidative stress, several lncRNAs act by increasing apoptosis like lncRNA BDNF-AS, FOXD3-AS1, Linc 00,963, and lncRNA ODRU (Wang X. et al., 2019) but antisense lncRNA natural BDNF-AS also regulates oxidative stress by reducing cell viability but its expression is inversely proportional to the synthesis of superoxide dismutase and catalase; Linc 00,963 also modulates the expression of the lncRNA FoxO3 in order to be able to attenuate renal fibrosis and oxidative stress during chronic renal failure; the lncRNA ODRU downregulates the level of Bcl2 expression through its interaction with the AKT and JNK signaling pathways as well as the PI4K alpha proteins. In diabetes, lncRNA SNHG16 increases the production of ROS and apoptosis *via* the increase in the level of expression of the KLF9 factor and that of miR-106a, MALAT 1, GAS5, CASC2, HOTAIR, MEG 3 are also implicated in this disorder (Chu et al., 2022). LncRNA MIAT is overexpressed following oxidation in the context of hypoxic pulmonary hypertension and its silencing by knockdown leads to the suppression of oxidative stress (Li, et al., 2020).

Furthermore, lncRNA LUCAT1 thanks to the increase in its level of expression, post-transcriptionally regulates the splicing and stability of NR4A2, leading to the activation of the production of inflammatory cytokines and interferons during inflammatory bowel and lung disease (Vierbuchen et al., 2022). This lncRNA is also upregulated in asthma and chronic obstructive pulmonary disease. lncRNA FAO regulates inflammation in macrophage cells followed by restoration of homeostasis by activating fatty acid oxidation *via* its interaction with the HADHB subunit of the mitochondrial trifunctional protein for FAO activation (Nakayama et al., 2020). Zhao et al., in 2022 showed that lncRNA Carl is an apoptosis-related lncRNA; during intestinal inflammation, the expression or overexpression of the lncRNA Carl leads to the expression of IL-1 beta and of the ptgs2 gene *via* its interaction with NF-kB P65; lncRNA CHRF leads to an increase in cytokine and oxidant levels following its overexpression, it also triggers the inflammation signaling pathway by activating MyD88 through miR-489; lncRNA FIRRE acts at the post-transcriptional level to regulate proinflammatory genes by interacting with hnRNPU proteins and by binding to the p56 subunit of NFkB; lncRNA PTPRE-AS1 acts *via* the MAPK/ERK1/2 signaling pathway to regulate inflammation *via* increasing and decreasing levels of TNF-alpha and IL-10 respectively; Long intergenic RNA not encoding proteins 638 (Linc00638) is implicated in several autoimmune diseases (Yanqiu et al., 2022). It regulates oxidative stress and inflammation especially by targeting inflammatory cytokines like IL-6, IL-17, IL-23 and regulating the Nrf2HO-1 pathway in rheumatoid arthritis (Sun et al., 2021). In human breast cancer, Small interfering RNA (siRNA) has been found to downregulate Notch-1 expression thereby causing an increase in chemosensitivity (Yang et al., 2010) while in prostate cancer, Notch silencing by siRNA increased chemoresistance by promoting cell growth inhibition (Ye et al., 2012). Piwi-interacting RNAs (piRNAs) have been reported to play a role in hepatocellular carcinoma, renal cell cancer, multiple myeloma (Yan et al., 2014), cardiovascular diseases (Yang J. et al., 2018), Alzheimer’s disease, and Parkinson’s disease (Qiu et al., 2017; Schulze et al., 2018).

We also observed that some lncRNAs can regulate oxidative stress and inflammation at the same time. The lncRNA Linc00638 acts by activating the Nrf2/HO-1 signaling pathways following an increase in its level of expression to inhibit oxidative stress and inflammation in rheumatoid arthritis (Sun et al., 2021); lncRNA OIP5-AS1 promotes inflammation and oxidative stress *via* up modulation of miR-128-3P expression to inhibit cyclin-dependent kinase 2A (Li et al., 2019).

## 6 Conclusion

We reviewed natural products that display biological effects on oxidative stress and inflammation-associated disorders *via* mechanisms involving the targeting of ncRNAs. Since this research axis is still emerging, there is a pressing need to shed more light on the mechanisms of these ncRNA-mediated biological activities which will advance the valorization of these natural compounds in the control and treatment of oxidative stress- and inflammation-associated disorders. Furthermore, an investigation is also warranted in the areas of the unknown ncRNAs-mediated mechanisms underlying natural products’ bioactivities and effects, ncRNAs involved in oxidative stress and inflammation-related disorders on which natural products have not been investigated as well as more natural products which may be implicated in common therapeutic remedies but whose effect has not been evaluated in the domain of oxidative stress and inflammation-related disorders.

## References

[B1] Abbasi B. Iqbal J. Mahmood T. Khalil A. T. Ali B. Kanwal S. (2018). Role of dietary phytochemicals in modulation of miRNA expression: Natural swords combating breast cancer. Asian Pac J. Trop. Med. 11, 501–509. 10.4103/1995-7645.242314

[B2] Abdullah M. A. (2020). Targeting micro-RNAs by natural products: A novel future therapeutic strategy to combat cancer. Am. J. Transl. Res. 12 (7), 3531–3556. Published online 2020 Jul 15, PMCID: PMC7407688. PMID: 32774718. 32774718 PMC7407688

[B3] Aghasafari P. George U. Pidaparti R. (2019). A review of inflammatory mechanism in airway diseases. Inflamm. Res. 68, 59–74. 10.1007/s00011-018-1191-2 30306206

[B4] Ahmad A. Sarkar S. H. Bitar B. Ali S. Aboukameel A. Sethi S. (2012). Garcinol regulates EMT and Wnt signaling pathways *in vitro* and *in vivo*, leading to anticancer activity against breast cancer cells. Mol. Cancer Ther. 11 (10), 2193–2201. PMID: 2282PMCID: PMC3836047. 10.1158/1535-7163.MCT-12-0232-T 22821148 PMC3836047

[B247] Ahmed M. A. E-B. (2014). “Sequence analysis of industrially important genes from trichoderma,” in Biotechnology and Biology of Trichoderma. Editors Gupta V. K. Schmoll M. Herrera-Estrella A. (Elsevier), 377–392.

[B5] Al Aameri R. F. H. Sheth S. Alanisi E. M. A. Borse V. Mukherjea D. Rybak L. P. (2017). Tonic suppression of PCAT29 by the IL-6 signaling pathway in prostate cancer: Reversal by resveratrol. PLoS One 12, e0177198. 10.1371/journal.pone.0177198 28467474 PMC5415196

[B6] Alharris E. Alghetaa H. Seth R. Chatterjee S. Singh N. P. Nagarkatti M. (2018). Resveratrol attenuates allergic asthma and associated inflammation in the lungs through regulation of miRNA-34a that targets FoxP3 in mice. Front. Immunol. 9, 2992. 10.3389/fimmu.2018.02992 30619345 PMC6306424

[B7] Alsahli M. A. Almatroodi S. A. Almatroudi A. Khan A. A. Anwar S. Almutary A. G. (2021). 6-Gingerol, a major ingredient of ginger attenuates diethylnitrosamine-induced liver injury in rats through the modulation of oxidative stress and anti-inflammatory activity. Mediat. Inflamm. 2021, 6661937. 10.1155/2021/6661937 PMC7837795 33531877

[B8] Aylan O. Metin O. (2015). Biochemistry of reactive oxygen and nitrogenspecies. London, UK: IntechOpen. 10.5772/61193

[B9] Ba L. Gao J. Chen Y. Qi H. Dong C. Pan H. (2019). Allicin attenuates pathological cardiac hypertrophy by inhibiting autophagy via activation of PI3K/Akt/mTOR and MAPK/ERK/mTOR signaling pathways. Phytomedicine 58, 152765. 10.1016/j.phymed.2018.11.025 31005720

[B10] Babajide O. A. Olajide T. A. Olayinka E. T. (2022). 6-gingerol attenuates pulmonary inflammation and oxidative stress in mice model of house dust mite-induced asthma. Adv. Redox Res. 5, 100036. 10.1016/j.arres.2022.100036

[B11] Bai X. Geng J. Li X. Wan J. Liu J. Zhou Z. M. (2018). Long noncoding RNA LINC01619 regulates MicroRNA-27a/forkhead box protein O1 and endoplasmic reticulum stress-mediated podocyte injury in diabetic nephropathy. Antioxidants Redox Signal. 29, 355–376. 10.1089/ars.2017.7278 29334763

[B12] Baldinu P. Cossu A. Manca A. Satta M. P. Sini M. C. Rozzo C. (2004). Identification of a novel candidate gene, CASC2, in a region of common allelic loss at chromosome 10q26 in human endometrial cancer. Buzz. Mutat. 23, 318–326. 10.1002/humu.20015 15024726

[B248] Banerjee J. Khanna S. Bhattacharya A. (2017). MicroRNA regulation of oxidative stress. Oxid. Med. Cell. Longev. 2017, 28721156. 10.1155/2017/2872156 PMC5684587 29312474

[B13] Beaver L. M. Kuintzle R. Buchanan A. Wiley M. W. Glasser S. T. Wong C. P. (2017). Long noncoding RNAs and sulforaphane: A target for chemoprevention and suppression of prostate cancer. J. Nutr. Biochem. 42, 72–83. 10.1016/j.jnutbio.2017.01.001 28131897 PMC5360475

[B14] Belaïch R. Boujraf S. (2016). Facteurs inflammatoires et stress oxydant chez les hémodialysés: Effets et stratégies thérapeutiques. Médecine Des. Mal. Métaboliques 10, 38–42. 10.1016/s1957-2557(16)30009-8

[B246] Bladé C. Baselga-Escudero L. Salvadó M. J. Arola-Arnal A. (2013). miRNAs, polyphenols, and chronic disease. Mol. Nutr. Food Res. 57 (1), 58–70. 10.1002/mnfr.201200454 23165995

[B15] Bowen D. Joong S. S. (2016). Targeting epithelial–mesenchymal transition (EMT) to overcome drug resistance in cancer. 10.3390/molecules21070965 PMC6273543 27455225

[B16] Brieger K. Schiavone S. Miller F. J. Jr Krause K. H. (2012). Reactive oxygen species: From health to disease. Swiss Med. Wkly. 142, 13659. 10.4414/smw.2012.13659 22903797

[B17] Calle M. C. Fernandez M. L. (2012). Inflammation and type 2 diabetes. Diabetes Metab. 38 (3), 183–191. 10.1016/j.diabet.2011.11.006 22252015

[B18] Carpi S. Polini B. Manera C. Maria D. Salsano J. E. Macchia M. (2020). MiRNA modulation and antitumor activity by the extra-virgin olive oil polyphénol Oleacein in human melanoma cells. Front. Pharmacol. 11, 574317. 10.3389/fphar.2020.574317 33071785 PMC7539365

[B19] Castellon X. Bogdanova V. (2016). Chronic inflammatory diseases and endothelial dysfunction. Aging Dis. 7 (1), 81–89. PMID: 26815098. 10.14336/AD.2015.0803 26815098 PMC4723236

[B20] Cesmeli S. Goker Bagca B. Caglar H. O. Ozates N. P. Gunduz C. Biray Avci C. (2022). Combination of resveratrol and BIBR1532 inhibits proliferation of colon cancer cells by repressing expression of LncRNAs. Med. Oncol. 39, 12–10. 10.1007/s12032-021-01611-w 34779924

[B21] Chang W. (2017). Non-coding RNAs and berberine: A new mechanism of its anti-diabetic activities. Eur. J. Pharmacol. 795, 8–12. 10.1016/j.ejphar.2016.11.055 27915042

[B22] Charles R. Lalith J. Eichhorn P. J. A. (2018). Platforms for investigating LncRNA functions. Oak Brook, Illinois, United States: SLAS TECHNOLOGY: Translating Life Sciences Innovation, 247263031878063. 10.1177/2472630318780639

[B23] Chaves S. K. Feitosa C. M. da S Araújo L. (2016). Alkaloids pharmacological activities - prospects for the development of phytopharmaceuticals for neurodegenerative diseases. Curr. Pharm. Biotechnol. 17 (7), 629–635. 10.2174/138920101707160503201541 26718919

[B24] Chen D. Daniel K. G. Kuhn D. J. Kazi A. Bhuiyan M. Li L. (2004). Green tea and tea polyphenols in cancer prevention. Front. Biosci. 9, 2618–2631. 10.2741/1421 15358585

[B25] Chen X. Tan X. R. Li S. J. Zhang X. X. (2019). LncRNA NEAT1 promotes hepatic lipid accumulation via regulating miR-146a-5p/ROCK1 in nonalcoholic fatty liver disease. Life Sci. 235, 116829. 10.1016/j.lfs.2019.116829 31484042

[B26] Chen Y. Huang C. Zhu S. Y. Zou H. C. Xu C. Y. Chen Y. X. (2021a). Overexpression of HOTAIR attenuates Pi-induced vascular calcification by inhibiting Wnt/β-catenin through regulating miR-126/Klotho/SIRT1 axis. Mol. Cell. Biochem. 476, 3551–3561. 10.1007/s11010-021-04164-8 34014438

[B27] Chen Y. Xu H. Liu C. Gu M. Zhan M. Chen Q. (2021b). LncRNA DIO3OS regulated by TGF-β1 and resveratrol enhances epithelial mesenchymal transition of benign prostatic hyperplasia epithelial cells and proliferation of prostate stromal cells. Transl. Androl. Urol. 10, 643–653. 10.21037/tau-20-1169 33718067 PMC7947439

[B28] Cheng J. Chen T. Li P. Wen J. Pang N. Zhang L. (2018). Sodium tanshinone IIA sulfonate prevents lipopolysaccharide-induced inflammation via suppressing nuclear factor-κB signaling pathway in human umbilical vein endothelial cells. Can. J. Physiol. Pharmacol. 96, 26–31. 10.1139/cjpp-2017-0023 28658584

[B29] Chu P. M. Yu C. C. Tsai K. L. Hsieh P. L. (2022). Regulation of oxidative stress by long non-coding RNAs in vascular complications of diabetes. Life (Basel) 12 (2), 274. 10.3390/life12020274 35207562 PMC8877270

[B30] Cote B. Elbarbry F. Bui F. Su J. W. Seo K. Nguyen A. (2022). Mechanistic basis for the role of phytochemicals in inflammation-associated chronic diseases. Molecules 27 (3), 781. 10.3390/molecules27030781 35164043 PMC8838908

[B31] Coussens L. M. Werb Z. (2002). Inflammation and cancer. Nature 420, 860–867. 10.1038/nature01322 12490959 PMC2803035

[B32] Cui C. Liu Y. Gerloff D. Rohde C. Pauli C. Köhn M. (2021). NOP10 predicts lung cancer prognosis and its associated small nucleolar RNAs drive proliferation and migration. Oncogene 40, 909–921. 10.1038/s41388-020-01570-y 33288886 PMC7862062

[B33] Cui-Cui Z. Fang N. (2019). LncRNA NEAT1 promotes inflammatory response in sepsis-induced liver injury via the Let-7a/TLR4 axis. Int. Immunopharmacol. 75, 1567–5769. 10.1016/j.intimp.2019.105731 31344555

[B34] Daoqi Z. Shao M. Yang J. Fang M. Liu S. Lou D. (2020). Curcumin enhances radiosensitization of nasopharyngeal carcinoma via mediating regulation of tumor stem-like cells by a CircRNA network. J. Cancer. 11 (8), 2360–2370. 10.7150/jca.39511 32127962 PMC7052922

[B35] De Bacco F. Luraghi P. Medico E. Reato G. Girolami F. Perera T. (2011). Induction of MET by ionizing radiation and its role in radioresistance and invasive growth of cancer. Jnci J. Natl. Cancer Inst. 103, 645–661. 10.1093/jnci/djr093 21464397

[B36] Deg L. L. Chi Y. Y. Liu L. Huang N. S. Wang L. Wu J. (2016). LINC00978 predicts poor prognosis in breast cancer patients. Sci. Rep. 6, 37936. 10.1038/srep37936 27897214 PMC5126584

[B37] Deng Q. Wen R. Liu S. Chen X. Song S. Li X. (2020). Increased long noncoding RNA maternally expressed gene 3 contributes to podocyte injury induced by high glucose through regulation of mitochondrial fission. Cell. death Dis. 11, 814. 10.1038/s41419-020-03022-7 32994406 PMC7525535

[B38] Dhar S. Kumar A. Rimando A. M. Zhang X. Levenson A. S. (2015). Resveratrol and pterostilbene epigenetically restore PTEN expression by targeting oncomiRs of the miR-17 family in prostate cancer. Oncotarget 6, 27214–27226. 10.18632/oncotarget.4877 26318586 PMC4694984

[B39] Domitrović R. Potočnjak I. (2016). A comprehensive overview of hepatoprotective natural compounds: Mechanism of action and clinical perspectives. Arch. Toxicol. 90 (1), 39–79. PMID: 26377694. 10.1007/s00204-015-1580-z 26377694

[B40] Donath M. Y. (2021). Glucose or insulin, which is the culprit in patients with COVID-19 and diabetes? Cell. Metab. 33 (1), 2–4. PMID: 3324PMCID: PMC7685630. 10.1016/j.cmet.2020.11.015 33248018 PMC7685630

[B41] Dutta S. Mahalanobish S. Saha S. Ghosh S. Sil P. C. (2019). Natural products: An upcoming therapeutic approach to cancer. Food Chem. Toxicol. 128, 240–255. 10.1016/j.fct.2019.04.012 30991130

[B42] Dzobo K. (2022). The role of natural products as sources of therapeutic agents for innovative drug discovery. Compr. Pharmacol. 2022, 408–422. 10.1016/B978-0-12-820472-6.00041-4

[B43] Eddaikra A. Eddaikra N. (2021). ‘Endogenous enzymatic antioxidant defense and pathologies’. Antioxidants - benefits, sources, mechanisms of action. London, UK: IntechOpen. 10.5772/intechopen.95504

[B44] Elham S. T. Chamasemani A. Firouzabadi N. Mousaei M. (2022). ncRNAs and polyphenols: new therapeutic strategies for hypertension. RNA Biol. 19 (1), 575–587. 10.1080/15476286.2022.2066335 35438046 PMC9037439

[B45] Enna S. J. Williams M. Barret J. F. Ferkany J. W. Kenakin T. Porsolt R. D. (2001). Current protocols in Pharmacology || natural products as a foundation for drug discovery. 10.1002/0471141755.ph0911s46

[B46] Erdmann V. A. Barciszewska M. Z. Szymanski M. Hochberg A. de Groot N. Barciszewski J. (2001). The non-coding RNAs as riboregulators. Nucleic Acids Res. 29, 189–193. 10.1093/nar/29.1.189 11125087 PMC29806

[B47] Eseberri I. Lasa A. Miranda J. Gracia A. Portillo M. P. (2017). Potential miRNA involvement in the anti-adipogenic effect of resveratrol and its metabolites. PLoS One 12, e0184875. 10.1371/journal.pone.0184875 28953910 PMC5617156

[B48] Esteller M. (2011). Non-coding RNAs in human disease. Nat. Rev. Genet. 12, 861–874. 10.1038/nrg3074 22094949

[B49] Fan G. Jiang X. Wu X. Fordjour P. A. Miao L. Zhang H. (2016). Anti-inflammatory activity of tanshinone IIA in LPS-stimulated RAW264.7 macrophages via miRNAs and TLR4-NF-κ B pathway. Inflammation 39, 375–384. 10.1007/s10753-015-0259-1 26639663

[B50] Fang Z. Y. Zhang M. Liu J. N. Zhao X. Zhang Y. Q. Fang L. (2021). Tanshinone IIA: A review of its anticancer effects. Front. Pharmacol. 11, 611087. 10.3389/fphar.2020.611087 33597880 PMC7883641

[B51] Fazal F. M. Han S. Parker K. R. Kaewsapsak P. Xu J. Boettiger A. N. (2019). Atlas of Subcellular RNA localization revealed by APEX-Seq. Cell. 178 (2), 473–490. 10.1016/j.cell.2019.05.027 31230715 PMC6786773

[B242] Fong N. Kim H. Zhou Y. Ji X. Qiu J. Saldi T. (2014). Pre-mRNA splicing is facilitated by an optimal RNA polymerase II elongation rate. Genes Dev. 28 (23), 2663–2676. 10.1101/gad.252106.114 25452276 PMC4248296

[B52] Gabriele P. Irrera N. Cucinotta M. Pallio G. Mannino F. Arcoraci V. (2017). Oxidative stress: Harms and benefits for human health. 10.1155/2017/8416763 PMC5551541 28819546

[B53] Gámez-Valero A. Guisado-Corcoll A. Herrero-Lorenzo M. Solaguren-Beascoa M. Martí E. (2020). Non-coding RNAs as sensors of oxidative stress in neurodegenerative diseases. Antioxidants (Basel) 9 (11), 1095. 10.3390/antiox9111095 33171576 PMC7695195

[B54] Gandy K. A. O. Zhang J. Nagarkatti P. Nagarkatti M. (2019). Resveratrol (3, 5, 4'-Trihydroxy-trans-Stilbene) attenuates a mouse model of multiple sclerosis by altering the miR-124/sphingosine kinase 1 Axis in encephalitogenic T cells in the brain. J. Neuroimmune Pharmacol. 14 (3), 462–477. PMID: 30941623. 10.1007/s11481-019-09842-5 30941623 PMC6900929

[B55] Gao J. Liang Z. Zhao F. Liu X. Ma N. (2022). Triptolide inhibits oxidative stress and inflammation via the microRNA-155-5p/brain-derived neurotrophic factor to reduce podocyte injury in mice with diabetic nephropathy. Bioengineered 13 (5), 12275–12288. 10.1080/21655979.2022.2067293 35603354 PMC9275869

[B56] Ge X. Xu B. Xu W. Xia L. Xu Z. Shen L. (2019). Long non-coding RNA GAS5 inhibits cell proliferation and fibrosis in diabetic nephropathy by sponging miR-221 and modulating SIRT1 expression. Aging 11, 8745–8759. 10.18632/aging.102249 31631065 PMC6834398

[B57] Geng W. Guo X. Zhang L. Ma Y. Wang L. Liu Z. (2018). Resveratrol inhibits proliferation, migration and invasion of multiple myeloma cells via NEAT1-mediated wnt/β-catenin signaling pathway. Biomed. Pharmacother. 107, 484–494. 10.1016/j.biopha.2018.08.003 30107344

[B58] Ghafouri-Fard S. Shoorei H. Taheri M. (2020). Non-coding RNAs are involved in the response to oxidative stress. Biomed. Pharmacother. 127, 110228. 10.1016/j.biopha.2020.110228 32559852

[B59] Giacco F. Brownlee M. (2010). Oxidative stress and diabetic complications. Circ. Res. 107 (9), 1058–1070. 10.1161/CIRCRESAHA.110.223545 21030723 PMC2996922

[B60] Giordo R. Wehbe Z. Posadino A. M. Erre G. L. Eid A. H. Mangoni A. A. (2022). Disease-associated regulation of non-coding RNAs by resveratrol: Molecular insights and therapeutic applications. Front. Cell. Dev. Biol. 10, 894305. 10.3389/fcell.2022.894305 35912113 PMC9326031

[B61] Gracia A. Miranda J. Fernández-Quintela A. Eseberri I. Garcia-Lacarte M. Milagro F. I. (2016). Involvement of miR-539-5p in the inhibition of de novo lipogenesis induced by resveratrol in white adipose tissue. Food Funct. 7, 1680–1688. 10.1039/c5fo01090j 26952965

[B62] Gusic M. Prokisch H. (2020). ncRNAs: New players in mitochondrial health and disease? Front. Genet. 11, 95. 10.3389/fgene.2020.00095 32180794 PMC7059738

[B63] He-da Z. Sun D. Mao L. Zhang J. Jiang L. Li J. (2015). MiR-139-5p inhibits the biological function of breast cancer cells by targeting Notch1 and mediates chemosensitivity to docetaxel. Biochem. Biophys. Res. Commun. 465 (4), 702–713. 10.1016/j.bbrc.2015.08.053 26299922

[B64] Heubach J. Monsior J. Deenen R. Niegisch G. Szarvas T. Niedworok C. (2015). The long noncoding RNA HOTAIR has tissue and cell type-dependent effects on HOX gene expression and phenotype of urothelial cancer cells. Mol. Cancer. 14, 108. 10.1186/s12943-015-0371-8 25994132 PMC4455698

[B65] Hong S. Youk T. Lee S. J. Kim K. M. Vajdic C. M. (2020). Bone metastasis and skeletal-related events in patients with solid cancer: A Korean nationwide health insurance database study. PLoS ONE 15 (7), e0234927. 10.1371/journal.pone.0234927 32678818 PMC7367479

[B66] Hu L. Zhao J. Liu Y. Liu X. Lu Q. Zeng Z. (2020). Geniposide inhibits proliferation and induces apoptosis of diffuse large B-cell lymphoma cells by inactivating the HCP5/miR-27b-3p/MET axis. Int. J. Med. Sci. 17 (17), 2735–2743. 10.7150/ijms.51329 33162801 PMC7645330

[B67] Hu M. Wang R. Li X. Fan M. Lin J. Zhen J. (2017). LncRNA MALAT1 is dysregulated in diabetic nephropathy and involved in high glucose-induced podocyte injury via its interplay with β-catenin. J. Cell. Mol. Med. 21, 2732–2747. 10.1111/jcmm.13189 28444861 PMC5661111

[B68] Huang C. Dong J. Jin X. Zhang D. Wang F., (2020). Intestinal anti-inflammatory effects of fuzi-ganjiang herb pair against DSS-induced ulcerative colitis in mice. J. Ethnopharmacol. 261, 112951. PMID: 32574670. 10.1016/j.jep.2020.112951 32574670

[B69] Huang L. Hu X. (2021). Molecular mechanisms and functions of lncRNAs in the inflammatory reaction of diabetes mellitus. Int. J. Endocrinol. 2021, 2550399. 10.1155/2021/2550399 34712322 PMC8548175

[B70] Huang Z. H. Du Yp. Wen Jt. Lu B. F. Zhao Y. (2022). snoRNAs: functions and mechanism in biological processes, and roles in tumor pathophysiology. Cell. Death Discov. 8 (1), 259. 10.1038/s41420-022-01056-8 35552378 PMC9098889

[B71] Hussain T. Tan B. Yin Y. Blachier F. Tossou M. C. Rahu N. (2016). Oxidative stress and inflammation: What polyphenols can do for us? Oxid. Med. Cell. Longev. 2016, 7432797. 10.1155/2016/7432797 27738491 PMC5055983

[B72] Imai-Sumida M. Majid S. Dasgupta P. Kulkarni P. Saini S. Bhagirath D. (2017). Abstract 3449: Genistein inhibits renal cancer progression through long non-coding RNA HOTAIR suppression. Cancer Res. 77, 3449. 10.1158/1538-7445.am2017-3449

[B73] Infante-Menéndez J. González-López P. Huertas-Lárez R. Gómez-Hernández A. Escribano Ó. (2023). Oxidative stress modulation by ncRNAs and their emerging role as therapeutic targets in atherosclerosis and non-alcoholic fatty liver disease. Antioxidants 12, 262. 10.3390/antiox12020262 36829822 PMC9952114

[B74] Irshad R. Husain M. (2021). Natural products in the reprogramming of cancer epigenetics. Toxicol. Appl. Pharmacol. 417, 115467. PMID: 33631231. 10.1016/j.taap.2021.115467 33631231

[B75] Iyer S. S. Pulskens W. P. Sadler J. J. Butter L. M. Teske G. J. Ulland T. K. (2009). Necrotic cells trigger a sterile inflammatory response through the Nlrp3 inflammasome. Proc. Natl. Acad. Sci. U. S. A. 106 (48), 20388–20393. 10.1073/pnas.0908698106 19918053 PMC2787135

[B76] Jabeen S. Hanifa M. S. Khan M. M. Qadri R. W. K. (2014). Natural products sources and their active compounds on disease prevention: A review. IJCBS 6, 76–83.

[B77] Javad H. Morvaridzadeh M. Maroufizadeh S. Akbari A. Yavari M. Amirinejad A. (2019). Omega-3 fatty acids supplementation and oxidative stress parameters: A systematic review and meta-analysis of clinical trials. Pharmacol. Res. 149, 104462. PMID: 31563611. 10.1016/j.phrs.2019.104462 31563611

[B245] Javaid A. Zahra D. Rashid F. Mashraqi M. Alzamami A. Khurshid M. (2022). Regulation of micro-RNA, epigenetic factor by natural products for the treatment of cancers: Mechanistic insight and translational association. Saudi J. Biol. Sci. 29 (6), 103255. 10.1016/j.sjbs.2022.03.005 35495735 PMC9052154

[B78] Jiabin Y. Zhu D. Liu S. Shao M. Liu Y. Li A. (2019). Curcumin enhances radiosensitization of nasopharyngeal carcinoma by regulating circRNA network. 10.1002/mc.23143 31793078

[B79] Ji‐An Z. Nie W. Dong L. Liu W. Wei W. (2021). A curcumin analog GL63 inhibits the malignant behaviors of hepatocellular carcinoma by inactivating the JAK2/STAT3 signaling pathway via the circular RNA zinc finger protein 83/microRNA‐324‐5p/cyclin‐dependent kinase 16 axis. 10.1111/jgh.15545 PMC8518784 33982329

[B80] Joye K. W. Ash S. L. Catignani G. L. (2004). Antioxidants and prevention of chronic disease. Crit. Rev. Food Sci. Nutr. 44 (4), 275–295. 10.1080/10408690490468489 15462130

[B81] Juan C. A. Pérez de la Lastra J. M. Plou F. J. Pérez-Lebeña E. (2021). The chemistry of reactive oxygen species (ROS) revisited: Outlining their role in biological macromolecules (DNA, lipids and proteins) and induced pathologies. Int. J. Mol. Sci. 22 (9), 4642. 10.3390/ijms22094642 33924958 PMC8125527

[B82] Kalalinia F. Karimi-Sani I. (2017). Anticancer properties of solamargine: A systematic review. Phytother. Res. 31 (6), 858–870. PMID: 28383149. 10.1002/ptr.5809 28383149

[B83] Kalhori M. R. Khodayari H. Khodayari S. Vesovic M. Jackson G. Farzaei M. H. (2021). Regulation of long non-coding RNAs by plant secondary metabolites: A novel anticancer therapeutic approach. 10.3390/cancers13061274 PMC8001769 33805687

[B84] Kamal F. Khan M. A. Lee-Smith W. Sharma S. Imam Z. Jowhar D. (2022). Outcomes of endoscopic submucosal dissection for treatment of superficial pharyngeal cancers: Systematic review and meta-analysis. Dig. Dis. Sci. 67, 3518–3528. 10.1007/s10620-021-07225-6 34505257

[B85] Kashyap D. Tuli H. S. Yerer M. B. Sharma A. Sak K. Srivastava S. (2021). Natural product-based nanoformulations for cancer therapy: Opportunities and challenges. Semin. Cancer Biol. 69, 5–23. 10.1016/j.semcancer.2019.08.014 31421264

[B86] Khansari N. Shakiba Y. Mahmoudi M. (2009). Chronic inflammation and oxidative stress as a major cause of age- related diseases and cancer. Recent Pat. Inflamm. Allergy Drug Discov. 3 (1), 73–80. 10.2174/187221309787158371 19149749

[B87] Khatkar A. Sharma K. K. (2019). Phenylpropanoids and its derivatives: Biological activities and its role in food, pharmaceutical and cosmetic industries. Crit. Rev. Food Sci. Nutr. 60, 1–21. 10.1080/10408398.2019.1653822 31456411

[B88] Khosravi A. R. Alder D. J. (2016). Chitin-induced airway epithelial cell innate immune responses are inhibited by carvacrol/thymol. PLoS ONE 11, e0159459. 10.1371/journal.pone.0159459 27463381 PMC4962986

[B89] Klebanoff S. J. (1980). Oxygen metabolism and the toxic properties of phagocytes. Ann. Intern. Med. 93, 480–489. 10.7326/0003-4819-93-3-480 6254418

[B90] Konovalova J. Gerasymchuk D. Parkkinen I. Chmielarz P. Domanskyi A. (2019). Interaction between microRNAs and oxidative stress in neurodegenerative diseases. 10.3390/ijms20236055 PMC6929013 31801298

[B91] Kornienko A. E. Dotter C. P. Guenzl P. M. Gisslinger H. Gisslinger B. Cleary C. (2016). Long non-coding RNAs display higher natural expression variation than protein-coding genes in healthy humans. Genome Biol. 17, 14. 10.1186/s13059-016-0873-8 26821746 PMC4731934

[B92] Kurek J. (2019). Alkaloids—their importance in nature and human life. London, UK: IntechOpen. Introductory Chapter: Alkaloids—Their Importance in Nature and for Human Life.

[B93] Kyriazis I. D. Hoffman M. Gaignebet L. Lucchese A. M. Markopoulou E. Palioura D. (2021). KLF5 is induced by FOXO1 and causes oxidative stress and diabetic cardiomyopathy. Circ. Res. 128, 335–357. 10.1161/CIRCRESAHA.120.316738 33539225 PMC7870005

[B94] Lagos-Quintana M. Rauhut R. Lendeckel W. Tuschl T. (2001). Identification of novel genes coding for small expressed RNAs. Science 294, 853–858. 10.1126/science.1064921 11679670

[B95] Latronico A. C. Silveira L. (2018). Genetic and epigenetic control of puberty. 10.1016/B978-0-12-801238-3.65226-1

[B96] Latruffe N. Lançon A. Frazzi R. Aires V. Delmas D. Michaille J.-J. (2015). Exploring new ways of regulation by resveratrol involving miRNAs, with emphasis on inflammation. Ann. N.Y. Acad. Sci. 1348, 97–106. 10.1111/nyas.12819 26190093

[B97] Lavieri R. Piccioli P. Carta S. Delfino L. Castellani P. Rubartelli A. (2014). TLR costimulation causes oxidative stress with unbalance of proinflammatory and anti-inflammatory cytokine production. J. Immunol. 192 (11), 5373–5381. 10.4049/jimmunol.1303480 24771848

[B98] Li H. Chen X. Liu J. Chen M. Huang M. Huang G. (2021). Ethanol extract of *Centella asiatica* alleviated dextran sulfate sodium-induced colitis: Restoration on mucosa barrier and gut microbiota homeostasis. J. Ethnopharmacol. 2021, 113445. 10.1016/j.jep.2020.113445 33022343

[B99] Li M. Ding W. Tariq M. A. Chang W. Zhang X. Xu W. (2018). A circular transcript of ncx1 gene mediates ischemic myocardial injury by targeting miR-133a-3p. Theranostic 8, 5855–5869. 10.7150/thno.27285 PMC6299442 30613267

[B100] Li Q. Pang L. Yang W. Liu X. Su G. Dong Y. (2018). Long non-coding RNA of myocardial infarction associated transcript (LncRNA-MIAT) promotes diabetic retinopathy by upregulating transforming growth factor-β1 (TGF-β1) signaling. Med. Sci. Monit. 24, 9497–9503. PMID: 3059PMCID: PMC6328291. 10.12659/MSM.911787 30595603 PMC6328291

[B101] Li X. Cao Q. Wang Y. Wang Y. (2019). Retracted article: LncRNA OIP5-AS1 contributes to ox-LDL-induced inflammation and oxidative stress through regulating the miR-128-3p/cdkn2a axis in macrophages. 10.1039/c9ra08322g PMC9076472 35541591

[B102] Li Y. Gao X. Wang Z. Liu W. Xu F. Hu Y. (2020). Circular RNA 4099 aggravates hydrogen peroxide-induced injury by down-regulating microRNA-706 in L02 cells. Life Sci. 241, 116826. 10.1016/j.lfs.2019.116826 31479678

[B103] Li Y. Wu X. Gao H. Jin J. M. Li A. X. Kim Y. S. (2015). Piwi-interacting RNAs (piRNAs) are dysregulated in renal cell carcinoma and associated with tumor metastasis and Cancer-specific survival. Mol. Med. 21, 381–388. 10.2119/molmed.2014.00203 25998508 PMC4534471

[B104] Li Y. Zhang X. Yang P. Zhang Z. Wu H. (2022). Preparation methods, structural characteristics, and biological activity of polysaccharides from Salvia miltiorrhiza: A review. J. Ethnopharmacol. 305, 116090. 10.1016/j.jep.2022.116090 36587878

[B105] Liang X. Xiao L. Luo Y. Xu J. (2020). Prevalence and risk factors of childhood hypertension in urban-rural areas of China: A cross-sectional study. Int. J. Hypertens. 2020, 2374231. 10.1155/2020/2374231 32454994 PMC7240786

[B106] Liang. J. Shen Y. C. Zhang X. Y. Chen C. Zhao H. Hu J. (2020). Circular RNA HIPK3 downregulation mediates hydrogen peroxide-induced cytotoxicity in human osteoblasts. Aging 12, 1159–1170. 10.18632/aging.102674 31955154 PMC7053588

[B107] Lippincott W. Wilkins (2016). Natural products: Naturally occurring compounds that are end products of secondary metabolism; often, they are unique compounds for particular organisms or classes of organisms.

[B108] Liu B. Sun L. Liu Q. Gong C. Yao Y. Lv X. (2015). A cytoplasmic NF-κB interacting long noncoding RNA blocks IκB phosphorylation and suppresses breast cancer metastasis. Cancer Cell. 27 (3), 370–381. PMID: 25759022. 10.1016/j.ccell.2015.02.004 25759022

[B109] Liu T. De Los Santos F, G. Phan S. H. (2017). The bleomycin model of pulmonary fibrosis. Methods Mol. Biol. 1627, 27–42. 10.1007/978-1-4939-7113-8_2 28836192

[B110] Liu Y. Chen X. Yao J. Kang J. (2019). Circular RNA ACR relieves high glucose-aroused RSC96 cell apoptosis and autophagy via declining microRNA-145-3p. J. Cell. Biochem. 122, 1252. 10.1002/jcb.29568 31886589

[B111] Lodish H. Berk A. Zipursky S. L. (2000). Molecular cell biology. 4th edition. New York: W. H. Freeman. Section 4.4, The Three Roles of RNA in Protein Synthesis.

[B112] Lopez-Mejia I. C. Fajas L. (2014). Cell cycle regulation of mitochondrial function. Curr. Opin. Cell. Biol. 33, 19–25. PMID: 25463842. 10.1016/j.ceb.2014.10.006 25463842

[B113] Lugrin J. Velin R. Roumen N. P. Liaudet L. (2014). The role of oxidative stress during inflammatory processes. Biol. Chem. 395 (2), 203–230. 10.1515/hsz-2013-0241 24127541

[B114] Ma D. Wei J. Chen S. Wang H. Ning L. Luo S. H. (2021). Fucoidan inhibits the progression of hepatocellular carcinoma via causing lncRNA LINC00261 overexpression. Front. Oncol. 11, 653902. 10.3389/fonc.2021.653902 33928038 PMC8078595

[B115] Mahmoud T. N. El-Maadawy W. H. Kandil Z. A. Khalil H. El-Fiky N. M. El Alfy T. S. M. A. (2021). Canna x generalis L.H. Bailey rhizome extract ameliorates dextran sulfate sodium-induced colitis via modulating intestinal mucosal dysfunction, oxidative stress, inflammation, and TLR4/NF-ҡB and NLRP3 inflammasome pathways. J. Ethnopharmacol. 269, 113670. PMID: 33301917. 10.1016/j.jep.2020.113670 33301917

[B116] Marseglia L. Manti S. D’Angelo G. Nicotera A. Parisi E. Di Rosa G. (2014). Oxidative stress in obesity: A critical component in human diseases. Int. J. Mol. Sci. 16 (1), 378–400. 10.3390/ijms16010378 25548896 PMC4307252

[B117] Ménézo Y. Entezami F. Lichtblau I. Cohen M. Belloc S. Brack M. (2012). Oxidative stress and fertility: False evidence and bad recipes. Gynecol. Obstet. Fertil. 40 (12), 787–796. PMID: 23177978. 10.1016/j.gyobfe.2012.09.032 23177978

[B118] Meng T. Qin W. Liu B. (2020). SIRT1 Antagonizes oxidative stress in diabetic vascular complication. front Endocrinol. 11, 568861. 10.3389/fendo.2020.568861 PMC7701141 33304318

[B119] Meng Y. Jin M. Yuan D. Zhao Y. Kong X. Guo X. (2022). Solamargine inhibits the development of hypopharyngeal squamous cell carcinoma by decreasing LncRNA HOXA11-as expression. Front. Pharmacol. 13, 887387. 10.3389/fphar.2022.887387 35903338 PMC9315292

[B120] Mezdour H. Hanfer M. Menad A. Ameddah S. (2017). Oxidative stress and its relationship with the emergence of various stomach damages. Batna J. Med. Sci. 4 (2), 145–148. 10.48087/BJMSra.2017.4204

[B121] Michael M. (2020). All you need to know about oxidative stress and inflammation.

[B122] Migdal C. Serres M. (2011). Reactive oxygen species and oxidative stress. Med. Sci. Paris. 27, 405–412. 10.1051/medsci/2011274017 21524406

[B123] Miranda J. L. Patricia J. E. Michele A. M. Moroney Hoult J. R. S. Halliwell B. (1991). Inhibition of mammalian 5-lipoxygenase and cyclo-oxygenase by flavonoids and phenolic dietary additives: Relationship to antioxidant activity and to iron ion-reducing ability. Biochem. Pharmacol. 42, 1673–1681. ISSN 0006-2952. 10.1016/0006-2952(91)90501-u 1656994

[B124] Mirzaei H. Masoudifar A. Sahebkar A. Zare N. Sadri Nahand J. Rashidi B. (2018). MicroRNA: A novel target of curcumin in cancer therapy. J. Cell. Physiol. 233 (4), 3004–3015. PMID: 28617957. 10.1002/jcp.26055 28617957

[B125] Mishra S. Verma S. S. Rai V. Awasthee N. Chava S. Hui K. M. (2019). Long non-coding RNAs are emerging targets of phytochemicals for cancer and other chronic diseases. Cell. Mol. Life Sci. 76 (10), 1947–1966. PMID: 3087PMCID: PMC7775409. 10.1007/s00018-019-03053-0 30879091 PMC7775409

[B126] Moreira P. I. Nunomura A. Nakamura M. Takeda A. Shenk J. C. Aliev G. (2008). Nucleic acid oxidation in Alzheimer disease. Free Radic. Biol. Med. 44, 1493–1505. 10.1016/j.freeradbiomed.2008.01.002 18258207

[B127] Nakayama Y. Fujiu K. Yuki R. Oishi Y. Morioka M. S. Isagawa T. (2020). A long noncoding RNA regulates inflammation resolution by mouse macrophages through fatty acid oxidation activation. Proc. Natl. Acad. Sci. U. S. A. 117 (25), 14365–14375. 10.1073/pnas.2005924117 32513690 PMC7322040

[B128] Nayansi J. Ryu J. J. Choi E. Kaushik N. K. (2017). Generation and Role of reactive oxygen and Nitrogen species induced by Plasma, lasers, chemical, Agents and other systems in dentistry. Oxidative Med. Cell. Longev. 2017, 7542540. 10.1155/2017/7542540 PMC5674515 29204250

[B129] Needham E. J. Helmy A. Zanier E. R. Jones J. L. Coles A. J. Menon D. K. (2019). The immunological response to traumatic brain injury. J. Neuroimmunol. 332, 112–125. 10.1016/j.jneuroim.2019.04.005 31005712

[B130] Niki E. (1997). “Free radicals in chemistry and biochemistry,” in Food and free radicals (New York: Hiramatsu Plenum Press).

[B131] Osbourn A. E. Lanzotti V. (2009). Plant-derived natural products introduction to the different classes of natural products. 10.1007/978-0-387-85498-4_1

[B132] Otsuka K. Yamamoto Y. Ochiya T. (2018). Regulatory role of resveratrol, a microRNA-controlling compound, in HNRNPA1 expression, which is associated with poor prognosis in breast cancer. Oncotarget 9 (37), 24718–24730. PMID: 2987PMCID: PMC5973863. 10.18632/oncotarget.25339 29872500 PMC5973863

[B133] Pahwa R. Goyal A. Jialal I. (2022). Chronic inflammation. Treasure Island (FL): StatPearls Publishing. Available from: https://www.ncbi.nlm.nih.gov/books/NBK493173. 29630225

[B134] Palmieri G. Paliogiannis P. Sini M. C. Manca A. Palomba G. Doneddu V. (2017). Long non-coding RNA CASC2 in human cancer. Writ. Rev. Oncol. Hematol. 111, 31–38. 10.1016/j.critrevonc.2017.01.003 28259293

[B135] Pan Y. Qian J. X. Lu S. Q. Chen J. W. Zhao X. D. Jiang Y. (2017). Protective effects of tanshinone IIA sodium sulfonate on ischemia-reperfusion-induced myocardial injury in rats. Iran. J. Basic. Med. Sci. 20, 308–315. 10.22038/ijbms.2017.8361 28392904 PMC5378969

[B136] Parasramka M. A. Ali S. Banerjee S. Deryavoush T. Sarkar F. H. Gupta S. (2013). Garcinol sensitizes human pancreatic adenocarcinoma cells to gemcitabine in association with microRNA signatures. Mol. Nutr. Food Res. 57 (2), 235–248. PMID: 23293055. 10.1002/mnfr.201200297 23293055

[B137] Pereira R. R. Guerra J. F. D. C. Lage N. N. Lopes J. M. M. Silva M. (2016). Açai (euterpe oleracea mart.) upregulates paraoxonase 1 gene expression and activity with concomitant reduction of hepatic steatosis in high-fat diet-fed rats. Oxid. Med. Cell. Longev. 2016, 8379105. 10.1155/2016/8379105 27642496 PMC5014968

[B138] Phurpa W. (2018). Therapeutic applications of natural products in herbal medicines, biodiscovery programs, and biomedicine. J. Biol. Act. Prod. Nat. 8 (1), 1–20. 10.1080/22311866.2018.1426495

[B139] Piergentili R. Basile G. Nocella C. Carnevale R. Marinelli E. Patrone R. (2022). Using ncRNAs as tools in cancer diagnosis and treatment-the way towards personalized medicine to improve patients' health. Int. J. Mol. Sci. 23 (16), 9353. 10.3390/ijms23169353 36012617 PMC9409241

[B140] Prasong S. Sriko J. Somanawat K. Chayanupatkul M. Klaikeaw N. Werawatganon D. (2022). Genistein mitigated oxidative stress, inflammation and apoptosis in acute L-arginine-induced pancreatitis in mice 2022. 10.1186/s12906-022-03689-9 PMC9351145 35927726

[B141] Qiang Z. Meng L. Yi C. Yu L. Chen W. Sha W. (2019). Curcumin regulates the miR-21/PTEN/Akt pathway and acts in synergy with PD98059 to induce apoptosis of human gastric cancer MGC-803 cells. J. Int. Med. Res. 47, 1288–1297. 10.1177/0300060518822213 30727807 PMC6421392

[B142] Qiu W. Guo X. Lin X. Yang Q. Zhang W. Zhang Y. (2017). Transcriptome-wide piRNA profiling in human brains of Alzheimer's disease. Neurobiol. Aging 57, 170–177. 10.1016/j.neurobiolaging.2017.05.020 28654860 PMC5542056

[B143] Ramanujam D. Schön A. P. Beck C. Vaccarello P. Felician G. Dueck A. (2021). MicroRNA-21–dependent macrophage-to-fibroblast signaling determines the cardiac response to pressure overload. Circulation 143 (15), 1513–1525. 10.1161/CIRCULATIONAHA.120.050682 33550817 PMC8032214

[B144] Rauf A. Imran M. Butt M. S. Nadeem M. Peters D. G. Mubarak M. S. (2018). Resveratrol as an anti-cancer agent: A review. Crit. Rev. Food Sci. Nutr. 58 (9), 1428–1447. 10.1080/10408398.2016.1263597 28001084

[B145] Ray P. D. Huang B. W. Tsuji Y. (2012). Reactive oxygen species (ROS) homeostasis and redox regulation in cellular signaling. Cell. Signal 24 (5), 981–990. 10.1016/j.cellsig.2012.01.008 22286106 PMC3454471

[B146] Rémy B. Minty M. Thomas C. Blasco V. (2022). Implication des bactéries orales et intestinales dans le décours des maladies cardio-métaboliques et du diabète de type 2. Médecine Des. Mal. Métaboliques 16, 121–133. 10.1016/j.mmm.2022.01.003

[B147] Ren D. Li F. Gao A. Cao Q. Liu Y. Zhang J. (2020). Hypoxia-induced apoptosis of cardiomyocytes is restricted by ginkgolide B-downregulated microRNA-29. Cell. Cycle 19 (10), 1067–1076. 10.1080/15384101.2020.1731651 32295500 PMC7217378

[B148] Ren T. Zhu Y. Xia X. Ding Y. Guo J. Kan J. (2017). *Zanthoxylum* alkylamides ameliorate protein metabolism disorder in STZ-induced diabetic rats. J. Mol. Endocrinol. 58 (3), 113–125. 10.1530/JME-16-0218 28100702 PMC5424265

[B149] Roy J. Galano J. M. Durand T. Jean-Yves L. G. Lee C. Y. (2017). Physiological role of reactive oxygen species as promoters of natural defenses. FASEB J. 31, 3729–3745. 10.1096/fj.201700170R 28592639

[B150] Ruiz-Manriquez L. M. Estrada-Meza C. Benavides-Aguilar J. Ledesma-Pacheco S. Torres-Copado A. Serrano‐Cano F. (2021). Phytochemicals mediated modulation of microRNAs and long non‐coding RNAs in cancer prevention and therapy. Phytotherapy Res. 36, 705–729. 10.1002/ptr.7338 34932245

[B151] Ryan K. A. Smith M. F. Jr Sanders M. K. Ernst P. B. (2004). Reactive oxygen and nitrogen species differentially regulate Toll-like receptor 4-mediated activation of NF-kappa B and interleukin-8 expression. Infect. Immun. 72, 2123–2130. 10.1128/iai.72.4.2123-2130.2004 15039334 PMC375203

[B152] Sadegh R. Rajani H. F. Mohammadkhani N. Ramirez-Coronel A. A. Maleki M. (2023). Long non-coding RNAs as novel targets for phytochemicals to cease cancer metastasis. Molecular 28, 987. 10.3390/molecules28030987 PMC9921150 36770654

[B153] Saghafi T. Taheri R. A. Parkkila S. Zolfaghari Emameh R. (2019). Phytochemicals as modulators of long non-coding RNAs and inhibitors of cancer-related carbonic anhydrases. Int. J. Mol. Sci. 20 (12), 2939. 10.3390/ijms20122939 31208095 PMC6627131

[B154] Salzano S. Checconi P. Hanschmann E. M. Lillig C. H. Bowler L. D. Chan P. (2014). Linkage of inflammation and oxidative stress via release of glutathionylated peroxiredoxin-2. which acts as a danger signal. Proc. Natl. Acad. Sci. U. S. A. 111 (33), 12157–12162. 10.1073/pnas.1401712111 25097261 PMC4143057

[B155] Sanjeev K. S. Arora S. Averett C. Singh S. Singh A. P. (2015). Modulation of MicroRNAs by phytochemicals in cancer: Underlying mechanisms and translational significance. BioMed Res. Int. 2015, 848710. Article ID 848710. 10.1155/2015/848710 25853141 PMC4380282

[B156] Schulze M. Sommer A. Plötz S. Farrell M. Winner B. Grosch J. (2018). Sporadic Parkinson's disease derived neuronal cells show disease-specific mRNA and small RNA signatures with abundant deregulation of piRNAs. Acta Neuropathol. Commun. 6 (1), 58. 10.1186/s40478-018-0561-x 29986767 PMC6038190

[B157] Shakir M. S. Li W. Morris N. L. Matter M. S. Colburn N. H. Kim Y. S. (2014). Resveratrol prevents tumorigenesis in mouse model of Kras activated sporadic colorectal cancer by suppressing oncogenic Kras expression. Carcinogenesis 35, 2778–2786. 10.1093/carcin/bgu209 25280562 PMC4247523

[B158] Shao J. Shi C. J. Li Y. Zhang F. W. Pan F. F. Fu W. M. (2020). LincROR mediates the suppressive effects of curcumin on hepatocellular carcinoma through inactivating wnt/β-catenin signaling. Front. Pharmacol. 11, 847. 10.3389/fphar.2020.00847 32714183 PMC7351502

[B159] Sharma A. Shahzad B. Rehman A. Bhardwaj R. Landi M. Zheng B. (2019). Response of phenylpropanoid pathway and the role of polyphenols in plants under abiotic stress. Molecules 24 (13), 2452. 10.3390/molecules24132452 31277395 PMC6651195

[B160] Sharma U. K. Sharma A. K. Pandey A. K. (2016). Medicinal attributes of major phenylpropanoids present in cinnamon. BMC Complement. Altern. Med. 16, 156. 10.1186/s12906-016-1147-4 27245453 PMC4888509

[B161] Shenouda S. M. Widlansky M. E. Chen K. Xu G. Holbrook M. Tabit C. E. (2011). Altered mitochondrial dynamics contributes to endothelial dysfunction in diabetes mellitus. Broadcast 124, 444–453. 10.1161/CIRCULATIONAHA.110.014506 PMC3149100 21747057

[B162] Shu L. Zhao H. Huang W. Hou G. Song G. Ma H. (2020). Resveratrol upregulates mmu-miR-363-3p via the PI3K-akt pathway to improve insulin resistance induced by a high-fat diet in mice. Dmso 13, 391–403. 10.2147/dmso.s240956 PMC7027849 32104036

[B163] Sifan S. Fang H. (2021). Curcumin inhibits ovarian cancer progression by regulating circ-PLEKHM3/miR-320a/SMG1 axis. 10.1186/s13048-021-00916-8 PMC8594156 34784955

[B164] Singla R. K. Dubey A. K. Garg A. Sharma R. K. Fiorino M. Ameen S. M. (2019). Natural polyphenols: Chemical classification, definition of classes, subcategories, and structures. J. AOAC Int. 102 (5), 1397–1400. PMID: 31200785. 10.5740/jaoacint.19-0133 31200785

[B165] Smith S. Tsai S. C. (2007). The type I fatty acid and polyketide synthases: A tale of two megasynthases. Nat. Product. Rep. 24 (5), 1041–1072. 10.1039/b603600g PMC2263081 17898897

[B166] Song J. Jun M. Ahn M.-R. Kim O. Y. (2016). Involvement of miR-Let7A in inflammatory response and cell survival/apoptosis regulated by resveratrol in THP-1 macrophage. Nutr. Res. Pract. 10, 377–384. 10.4162/nrp.2016.10.4,377 27478543 PMC4958639

[B167] Soto-Blanco B. (2022). “Chapter 12 - herbal glycosides in healthcare,” in Herbal biomolecules in healthcare applications. Editors Mandal S. C. Nayak A. K. Dhara A. K. (Cambridge, Massachusetts: Academic Press), 239–282.

[B168] Soudeh G. F. Shoorei H. Taheri M. (2020). Non-coding RNAs are involved in the response to oxidative stress. Biomed. Pharmacother. 127, 110228. 10.1016/j.biopha.2020.110228 32559852

[B169] Statello L. Guo C.-J. Chen L.-L. Huarte M. (2021). Gene regulation by long non-coding RNAs and its biological functions. Nat. Rev. Mol. Cell. Biol. 22 (22), 96–118. 10.1038/s41580-020-00315-9 33353982 PMC7754182

[B170] Su Q. Yao J. Sheng C. (2018). Geniposide attenuates LPS-induced injury via up-regulation of miR-145 in H9c2 cells. Inflammation 41, 1229–1237. 10.1007/S10753-018-0769-8 29611016

[B171] Subrata K. B. (2016). Does the interdependence between oxidative stress and inflammation explain the antioxidant paradox? Oxidative Med. Cell. Longev. 2016, 9. Article ID 5698931. 10.1155/2016/5698931 PMC4736408 26881031

[B172] Sun H. J. Hou B. Wang X. Zhu X. X. Li K. X. Qiu L. Y. (2016). Endothelial dysfunction and cardiometabolic diseases: Role of long non-coding RNAs. Life Sci. 167, 6–11. 10.1016/j.lfs.2016.11.005 27838210

[B173] Sun Y. Liu J. Xin L. Zhou Q. Chen X. Ding X. (2021). The low expression of long non-coding RNA Linc00638 contributes to the progression of rheumatoid arthritis by regulating inflammation and oxidative stress. J. South Med. Univ. 41 (7), 965–971doi. 10.12122/j.issn.1673-4254.2021.07.01 PMC8329686 34308844

[B174] Sun Z. Wang H. Wang J. Zhou L. Yang P. (2014). Chemical composition and anti-inflammatory, cytotoxic and antioxidant activities of essential oil from leaves of mentha piperita grown in China. PLoS ONE 9 (12), e114767. 10.1371/journal.pone.0114767 25493616 PMC4262447

[B175] Szyman’ski M. Barciszewski J. (2002). Beyond the proteome: Non-coding regulatory RNAs. Genome Biol. 3, reviews0005. 10.1186/gb-2002-3-5-reviews0005 12049667 PMC139358

[B241] Tabas I. Glass C. K. (2013). Anti-inflammatory therapy in chronic disease: Challenges and opportunities. Science 339 (6116), 166–172. 10.1126/science.1230720 23307734 PMC3608517

[B176] Tahamtan A. Teymoori-Rad M. Nakstad B. Salimi V. (2018). Anti-inflammatory MicroRNAs and their potential for inflammatory diseases treatment. Front. Immunol. 9, 1377. 10.3389/fimmu.2018.01377 29988529 PMC6026627

[B177] Tamgue O. Chia J. E. Brombacher F. (2021). Triptolide modulates the expression of inflammation-associated lncRNA-PACER and lincRNA-p21 in Mycobacterium tuberculosis-infected monocyte-derived macrophages. Front. Pharmacol. 12, 618462. 10.3389/fphar.2021.618462 33912039 PMC8071990

[B178] Tu J. Xing Y. Guo Y. Tang F. Guo L. Xi T. (2012). TanshinoneIIA ameliorates inflammatory microenvironment of colon cancer cells via repression of microRNA-155. Int. Immunopharmacol. 14 (4), 353–361. 10.1016/j.intimp.2012.08.015 22982040

[B179] Uchida S. Adams J. C. (2019). Roles of non-coding RNAs in human diseases [introductory editorial for the theme: Roles of non-coding RNAs in human diseases]. Am. J. Physiology-Cell Physiology. ajpcell. 317, C1–C2. 10.1152/ajpcell.00114.2019 PMC6689750 31091141

[B180] Valcheva-Kuzmanova S. Kuzmanov K. Tancheva S. Belcheva A. (2007). Hypoglycemic and hypolipidemic effects of Aronia melanocarpa fruit juice in streptozotocin-induced diabetic rats. Methods Find. Exp. Clin. Pharmacol. 29 (2), 101–105. 10.1358/mf.2007.29.2.1075349 17440626

[B181] Venkatadri R. Muni T. Iyer A. K. V. Yakisich J. S. Azad N. (2016). Role of apoptosis-related miRNAs in resveratrol-induced breast cancer cell death. Cell. Death Dis. 7, e2104. 10.1038/cddis.2016.6 26890143 PMC5399194

[B182] Veronique H. Christine M. (2012). Evidence for a protective effect of polyphenols-containing foods on cardiovascular health: An update for clinicians. Bethesda, Maryland, United States: NCBI. 10.1177/2040622311430006 PMC3513903 23251771

[B183] Vierbuchen T. Agarwal S. Johnson J. L. Galia L. Lei X. Stein K. (2022). The lncRNA LUCAT1 is elevated in inflammatory disease and restrains inflammation by regulating the splicing and stability of NR4A2. Proc. Natl. Acad. Sci. U. S. A. 120 (1), e2213715120. 10.1073/pnas.2213715120 36577072 PMC9910463

[B184] Vinciguerra M. Sgroi A. Veyrat-Durebex C. Rubbia-Brandt L. Buhler L. H. Foti M. (2009). Unsaturated fatty acids inhibit the expression of tumor suppressor phosphatase and tensin homolog (PTEN) via microRNA-21 up-regulation in hepatocytes. Hepatology 49 (4), 1176–1184. 10.1002/hep.22737 19072831

[B185] Visioli F. Giordano E. Nicod N. M. Dávalos A. (2012). Molecular targets of omega 3 and conjugated linoleic Fatty acids - "micromanaging" cellular response. Front. Physiol. 3, 42. 10.3389/fphys.2012.00042 22393325 PMC3289952

[B186] Wang B. Trayhurn P. (2006). Acute and prolonged effects of TNF-alpha on the expression and secretion of inflammation-related adipokines by human adipocytes differentiated in culture. Pflüg. Arch. 452, 418–427. 10.1007/s00424-006-0055-8 16586095

[B187] Wang B. Wang Y. Xu K. Zeng Z. Xu Z. Yue D. (2021). Resveratrol alleviates sepsis-induced acute kidney injury by deactivating the lncRNA MALAT1/MiR-205 axis. cejoi 46, 295–304. 10.5114/ceji.2021.109195 PMC8574118 34764801

[B188] Wang L. Zhang R. Chen J. Wu Q. Kuang Z. (2017). Baicalin protects against TNF-α-induced injury by down-regulating miR-191a that targets the tight junction protein ZO-1 in IEC-6 cells. Biol. Pharm. Bull. 40 (4), 435–443. PMID: 28111380. 10.1248/bpb.b16-00789 28111380

[B189] Wang R. Ma Z. Feng L. Yang Y. Tan C. Shi Q. (2018). LncRNA MIR31HG targets HIF1A and P21 to facilitate head and neck cancer cell proliferation and tumorigenesis by promoting cell-cycle progression. Mol. Cancer 17 (1), 162. 10.1186/s12943-018-0916-8 30458787 PMC6247607

[B190] Wang W. Yang N. Yang Y. H. Wen R. Liu C. F. (2021). Non-coding RNAs: Master regulators of inflammasomes in inflammatory diseases. J. Inflamm. Res. 14, 5023–5050. 10.2147/JIR.S332840 34616171 PMC8490125

[B191] Wang W. H. Chen J. Zhang B. R. Lu S. J. Wang F. Peng L. (2018). Curcumin inhibits proliferation and enhances apoptosis in A549 cells by downregulating lncRNA UCA1. Pharmazie 73 (7), 402–407. 10.1691/ph.2018.8402 30001775

[B192] Wang X. Shen C. Zhu J. Shen G. Li Z. Dong J. (2019). Long noncoding RNAs in the regulation of oxidative stress. Oxid. Med. Cell. Longev. 2019, 1318795. 10.1155/2019/1318795 30911342 PMC6398004

[B243] Wang X. Wang W. HuangFu W. Liu Z. Zhao F. (2022). LncRNA HOTAIR facilitates high glucose-induced mesangial cell proliferation, fibrosis and oxidative stress in diabetic nephropathy via regulating miR-147a/WNT2B axis. Diabetol. Metab. Syndr. 14 (33). 10.1186/s13098-022-00802-3 PMC8864868 35193668

[B193] Wang Y. Yang S. H. Zhong K. Jiang T. Zhang M. Kwan H. Y. (2020). Network pharmacology-based strategy for the investigation of the anti-obesity effects of an ethanolic extract of *Zanthoxylum bungeanum*Maxim. Front. Pharmacol. 11, 572387. 10.3389/fphar.2020.572387 33364948 PMC7751641

[B194] Wang Y. Zhao R. Liu W. Wang Z. Rong J. Long X. (2019). Exosomal circHIPK3 released from hypoxia-pretreated cardiomyocytes regulates oxidative damage in cardiac microvascular endothelial cells via the miR-29a/IGF-1 pathway. Oxidative Med. Cell. Longev. 2019, 7954657. 10.1155/2019/7954657 PMC6915129 31885817

[B195] Wei X. Zhang L. Geng Y. Liu Y. Zhang N. (2020). Long noncoding RNA GAS5 promotes microglial inflammatory response in Parkinson's disease by regulating NLRP3 pathway through sponging miR-223-3p. Int. Immunopharmacol. 85, 106614. ISSN 1567-5769. 10.1016/j.intimp.2020.106614 32470877

[B196] Wu F. Cui L. (2017). Resveratrol suppresses melanoma by inhibiting NF-κB/miR-221 and inducing TFG expression. Arch. Dermatol Res. 309, 823–831. 10.1007/s00403-017-1784-6 28936555

[B197] Xi W. Pan Y. Yuan F. Zhang J. Xie Me Yang F. (2020). The biogenesis and functions of piRNAs in human diseases. Mol. Ther. - Nucleic Acids 21, 108–120. ISSN 2162-2531. 10.1016/j.omtn.2020.05.023 32516734 PMC7283962

[B198] Xiao X. Shi D. Liu L. Wang J. Xie X. Kang T. (2011). Quercetin suppresses cyclooxygenase-2 expression and angiogenesis through inactivation of P300 signaling. PLoS One 6 (8), e22934. 10.1371/journal.pone.0022934 21857970 PMC3152552

[B199] Xiaoqing X. Zhang X. Zhang Y. Wang Z. (2021). Curcumin suppresses the malignancy of non-small cell lung cancer by modulating the circ-PRKCA/miR-384/ITGB1 pathway. 10.1016/j.biopha.2021.111439 33684690

[B200] Xie C. Kang J. Li Z. Schauss A. G. Badger T. M. Nagarajan S. (2012). The açaí flavonoid velutin is a potent anti-inflammatory agent: Blockade of LPS-mediated TNF-α and IL-6 production through inhibiting NF-κB activation and MAPK pathway. J. Nutr. Biochem. 23, 1184–1191. PMID: 22137267. 10.1016/j.jnutbio.2011.06.013 22137267

[B201] Xie C. Wu W. Tang A. Luo N. Tan Y. (2019). lncRNA GAS5/miR-452-5p reduces oxidative stress and pyroptosis of high-glucose-stimulated renal tubular cells. Diabetes, Metabolic Syndrome Obes. Targets Ther. 12, 2609–2617. 10.2147/DMSO.S228654 PMC6910862 31849505

[B202] Xie L. Tang H. Song J. Long J. Zhang L. Li X. (2019). Chrysophanol: A review of its pharmacology, toxicity and pharmacokinetics. J. Pharm. Pharmacol. 71, 1475–1487. 10.1111/jphp.13143 31373015

[B203] Xie W. Chang W. Wang X. Liu F. Wang X. Yuan D. (2022). Allicin inhibits osteosarcoma growth by promoting oxidative stress and autophagy via the inactivation of the lncRNA MALAT1-miR-376a-wnt/β-catenin signaling pathway. Oxid. Med. Cell. Longev. 2022, 4857814. 10.1155/2022/4857814 35783190 PMC9249524

[B204] Xu X. Zhang Y. (2022). Regulation of oxidative stress by long non-coding RNAs in central nervous system disorders. Front. Mol. Neurosci. 15, 931704. 10.3389/fnmol.2022.931704 35782387 PMC9241987

[B205] Xu X. H. Ding D. F. Yong H. J. Dong C. L. You N. Ye X. L. (2017). Resveratrol transcriptionally regulates miRNA-18a-5p expression ameliorating diabetic nephropathy via increasing autophagy. Eur. Rev. Med. Pharmacol. Sci. 21, 4952–4965. 29164562

[B206] Xuan Y. Gao Y. Huang H. Wang X. Cai Y. Luan Q. X. (2017). Tanshinone IIA attenuates atherosclerosis in apolipoprotein E knockout mice infected with porphyromonas gingivalis. Ignition 40, 1631–1642. 10.1007/s10753-017-0603-8 28646427

[B207] Yan H. Wu Q. L. Sun C. Y. Ai L. S. Deng J. Zhang L. (2014). piRNA-823 contributes to tumorigenesis by regulating de novo DNA methylation and angiogenesis in multiple myeloma. Leukemia 29 (1), 196–206. PMID: 24732595. 10.1038/leu.2014.135 24732595

[B208] Yan X. Hu Z. Feng Y. Hu X. Yuan J. Zhao S. D. (2015). Comprehensive genomic characterization of long non-coding RNAs across human cancers. Cancer Cell. 28 (4), 529–540. 10.1016/j.ccell.2015.09.006 26461095 PMC4777353

[B209] Yang J. Xue F. T. Li Y. Y. Liu W. Zhang S. (2018). Exosomal piRNA sequencing reveals differences between heart failure and healthy patients. Eur. Rev. Med. Pharmacol. Sci. 22 (22), 7952–7961. 10.26355/eurrev_201811_16423 30536343

[B210] Yang X. Jiang J. Zhang C. Li Y. (2019). Baicalein restrains proliferation, migration, and invasion of human malignant melanoma cells by down-regulating colon cancer associated transcript-1. Braz. J. Med. Biol. Res. 52, 8934. 10.1590/1414-431X20198934 PMC6886380 31778440

[B211] Yang Y. Q. Yan X. T. Wang K. Tian R. M. Lu Z. Y. Wu L. L. (2018). Triptriolide alleviates lipopolysaccharide-induced liver injury by Nrf2 and NF-κB signaling pathways. Front. Pharmacol. 9, 999. 10.3389/fphar.2018.00999 30210350 PMC6124152

[B212] Yang Z. Li H. Sun J. Gao J. Liu W. Li B. (2010). DC-chol/DOPE cationic liposomes: A comparative study of the influence factors on plasmid pDNA and siRNA gene delivery. Int. J. Pharm. 390, 198–207. ISSN 0378-5173. 10.1016/j.ijpharm.2010.01.035 20116418

[B213] Yanqiu S. Jian L. Jianting W. Dan H. Qin Z. Xianheng Z. (2022). Overexpression of the long non-coding RNA LINC00638 inhibits inflammation and oxidative stress in rheumatoid arthritis fibroblast-like synoviocytes by regulating the Nrf2-HO1 pathway. Immun. Inflamm. Dis. 10, e663. 10.1002/iid3.663 35759235 PMC9208282

[B214] Yao S. Gao M. Wang Z. Wang W. Zhan L. Wei B. (2021). Upregulation of MicroRNA-34a sensitizes ovarian cancer cells to resveratrol by targeting bcl-2. Yonsei Med. J. 62, 691–701. 10.3349/ymj.2021.62.8.691 34296546 PMC8298871

[B215] Yasuda M. Ohzeki Y. Shimizu S. Naito S. Ohtsuru A. Yamamoto T. (1999). Stimulation of *in vitro* angiogenesis by hydrogen peroxide and the relation with ETS-1 in endothelial cells. Life Sci. 64, 249–258. 10.1016/s0024-3205(98)00560-8 10027759

[B216] Ye Q. F. Zhang Y. C. Peng X. Q. Long Z. Ming Y. Z. He L. Y. (2012). siRNA-mediated silencing of Notch-1 enhances docetaxel induced mitotic arrest and apoptosis in prostate cancer cells. Asian Pac J. Cancer Prev. 13 (6), 2485–2489. PMID: 22938409. 10.7314/apjcp.2012.13.6.2485 22938409

[B217] Yongzhong W. Lu J. Antony S. Juhasz A. Han L. Jiang G. (2013). Activation of TLR4 is required for the synergistic induction of dual oxidase 2 and dual oxidase A2 by IFN-γ and lipopolysaccharide in human pancreatic cancer cell lines. J. Immunol. 190 (4), 1859–1872. 10.4049/jimmunol.1201725 23296709 PMC3563939

[B218] Yu X. Tang W. Yang Y. Tang L. Dai R. Pu B. (2018). Long noncoding RNA NKILA enhances the anti-cancer effects of baicalein in hepatocellular carcinoma via the regulation of NF-κB signaling. Chem. Interact. 285, 48–58. 10.1016/j.cbi.2018.02.027 29481769

[B219] Yu Y. Sarkar F. H. Majumdar A. P. N. (2013). Down-regulation of miR-21 induces differentiation of chemoresistant colon cancer cells and enhances susceptibility to therapeutic regimens. Transl. Oncol. 6 (2), 180–186. 10.1593/tlo.12397 23544170 PMC3610548

[B220] Yuan K. Li X. Lu Q. Zhu Q. Jiang H. Wang T. (2019). Application and mechanisms of triptolide in the treatment of inflammatory diseases-A review. Front. Pharmacol. 10, 1469. PMID: 31866868. 10.3389/fphar.2019.01469 31866868 PMC6908995

[B221] Zha F. Qu X. Tang B. Li J. Wang Y. Zheng P. (2019). Long non-coding RNA MEG3 promotes fibrosis and inflammatory response in diabetic nephropathy via miR-181a/Egr-1/TLR4 axis. Aging 11, 3716–3730. 10.18632/aging.102011 31195367 PMC6594792

[B222] Zhang C. C. Niu F. (2019). LncRNA NEAT1 promotes inflammatory response in sepsis-induced liver injury via the Let-7a/TLR4 axis. Int. Immunopharmacol. 75, 105731. 10.1016/j.intimp.2019.105731 31344555

[B223] Zhang H. Liu X. Liu Y. Liu J. Gong X. Li G. (2022). Crosstalk between regulatory non-coding RNAs and oxidative stress in Parkinson's disease. Front. Aging Neurosci. 14, 975248. 10.3389/fnagi.2022.975248 36016854 PMC9396353

[B224] Zhang H. Yan Y. Hu Q. Zhang X. (2021). LncRNA MALAT1/microRNA let-7f/KLF5 axis regulates podocyte lesions in diabetic nephropathy. Sci. Life 266, 118794. 10.1016/j.lfs.2020.118794 33232688

[B225] Zhang J. Chen M. Zhai Y. Fu Y. (2020). Retracted: HOTAIR regulates lipopolysaccharide‐induced inflammatory response in hepatocytes. J. Cell. Physiol. 235 (5), 4247–4255. 10.1002/jcp.29301 31621909

[B226] Zhang L. Ma R. Gao M. Zhao Y. Lv X. Zhu W. (2020a). SNORA72 activates the Notch1/c-Myc pathway to promote stemness transformation of ovarian cancer cells. Front. Cell. Dev. Biol. 8, 583087. 10.3389/fcell.2020.583087 33224949 PMC7669759

[B227] Zhang L. Zhao S. Zhu Y. (2020b). Long noncoding RNA growth arrest-specific transcript 5 alleviates renal fibrosis in diabetic nephropathy by downregulating matrix metalloproteinase 9 through recruitment of enhancer of zeste homolog 2. FASEB J. 34, 2703–2714. 10.1096/fj.201901380RR 31916627

[B228] Zhang R. Ma X. Jiang L. Xia W. Li H. Zhao N. (2021). Decreased lncRNA SNHG16 accelerates oxidative stress induced pathological angiogenesis in human retinal microvascular endothelial cells by regulating miR-195/mfn2 Axis. Curr. Pharm. Des. 27, 3047–3060. 10.2174/1381612827666210202141541 33530902

[B229] Zhang S. Long F. Lin H. Wang X. Jiang G. Wang T. (2021). Regulatory roles of phytochemicals on circular RNAs in cancer and other chronic diseases. Pharmacol. Res. 174, 105936. 10.1016/j.phrs.2021.105936 34653635

[B230] Zhang W. Lu C. Cai S. Feng Y. Shan J. Di L. (2022). Aconiti lateralis radix praeparata as potential anticancer herb: Bioactive compounds and molecular mechanisms. Front. Pharmacol. 13, 870282. PMID: 35662730. 10.3389/fphar.2022.870282 35662730 PMC9158441

[B231] Zhang X.-L. Zhu H.-Q. Zhang Y. Zhang N. Y. Jiao J.-S. Xing X.-Y. (2020). LncRNA CASC2 regulates high glucose-induced proliferation, extracellular matrix accumulation and oxidative stress of human mesangial cells via miR-133b/FOXP1 axis. Eur. Rev. Med. Pharmacol. Sci. 24, 802–812. 10.26355/eurrev_202001_20063 32016985

[B232] Zhang Y. Wang M. Dong H. Yu X. Zhang J. (2018). Anti-hypoglycemic and hepatocyte-protective effects of hyperoside from Zanthoxylum bungeanum leaves in mice with high-carbohydrate/high-fat diet and alloxan-induced diabetes. Int. J. Mol. Med. 41 (1), 77–86. 10.3892/ijmm.2017.3211 29115390 PMC5746319

[B233] Zhao H. Ming T. Tang S. Ren S. Yang H. Liu M. (2022). Wnt signaling in colorectal cancer: Pathogenic role and therapeutic target. Mol. Cancer 21 (1), 144. 10.1186/s12943-022-01616-7 35836256 PMC9281132

[B234] Zhao Q. Pang G. Yang L. Chen S. Xu R. Shao W. (2021). Long noncoding RNAs regulate the inflammatory responses of macrophages. Cells 11 (1), 5. 10.3390/cells11010005 35011565 PMC8750547

[B235] Zheng W. Li T. Wei J. Zhang Y. Zuo Q. Lin Y. (2021). Identification of miR-145 as a regulator of the cardiomyocyte inflammatory response and oxidative stress under hyperglycemia. Exp. Ther. Med. 21, 467. 10.3892/etm.2021.9898 33763154 PMC7983182

[B236] Zhenping G. Dan H. (2021). lncRNA GAS 5-mediated miRNA-23a-3p promotes inflammation and cell apoptosis by targeting TLR4 in a cell model of sepsis. 10.3892/mmr.2021.12149 PMC8138517 33982771

[B237] Zhou J. Zhao Y. Li Z. Zhu M. Wang Z. Li Y. (2020). miR-103a-3p regulates mitophagy in Parkinson's disease through Parkin/Ambra1 signaling. Pharmacol. Res. 160, 105197. PMID: 32942015. 10.1016/j.phrs.2020.105197 32942015

[B238] Zhou Y. Wang F. Li W. Hong H. Chen J. Tian Y. (2018). Protective effects of microRNA-330 on namyloid B-protein production, oxidative stress, and mitochondrial dysfunction in Alzheimer’s disease by targeting VAV1 via the MAPK signaling pathway. 10.1002/jcb.26700 29369410

[B239] Zhou Z. Zhu Y. Gao G. Zhang Y. (2019). Long noncoding RNA SNHG16 targets miR-146a-5p/CCL5 to regulate LPS-induced WI-38 cell apoptosis and inflammation in acute pneumonia. Life Sci. 228, 189–197. 10.1016/j.lfs.2019.05.008 31071307

[B240] Zhu L. Mou Q. Wang Y. Zhu Z. Cheng M. (2020). Resveratrol contributes to the inhibition of liver fibrosis by inducing autophagy via the microRNA-20a-mediated activation of the PTEN/PI3K/AKT signaling pathway. Int. J. Mol. Med. 46, 2035–2046. 10.3892/ijmm.2020.4748 33125088 PMC7595670

